# Minimalistic Peptide Nanocarriers for Multiple Cancer Drugs

**DOI:** 10.1021/acsabm.5c01234

**Published:** 2025-10-06

**Authors:** Anastasia Vlachou, Om Shanker Tiwari, Sonika Chibh, Jake R. Remmert, Ehud Gazit, Phanourios Tamamis

**Affiliations:** † Artie McFerrin Department of Chemical Engineering, 14736 Texas A&M University, College Station, Texas 77843-3122, United States; ‡ The Shmunis School of Biomedicine and Cancer Research, George S. Wise Faculty of Life Sciences, 26745 Tel Aviv University, Tel Aviv 6997801, Israel; § Department of Materials Science and Engineering, Iby and Aladar Fleischman Faculty of Engineering, Tel Aviv University, Tel Aviv 6997801, Israel; ∥ Sagol School of Neuroscience, Tel Aviv University, Tel Aviv 6997801, Israel; ⊥ Department of Materials Science and Engineering, Texas A&M University, College Station, Texas 77843-3003, United States

**Keywords:** minimalism, short peptides, cancer drugs, co-assembly, nanocarriers

## Abstract

The co-assembly of minimalistic peptides with cancer drugs, leading to the formation of nanocarriers for drug delivery, comprises a promising direction in chemotherapeutics. We computationally designed fluorescent minimalistic four-residue peptide nanocarriers for multiple cancer drugs: Epirubicin, Doxorubicin, Methotrexate, Mitomycin-C, 5-Fluorouracil, Camptothecin, and Cyclophosphamide. The optimally designed resulting nanocarriers formed by FFWH have notable drug encapsulation properties for the drugs investigated, according to both computational and experimental studies. Additionally, the nanocarriers possess biocompatibility, enhanced fluorescence, and uptake into HeLa cells using live cell confocal microscopic images. Our simulations demonstrate how the same peptide can efficiently be used to encapsulate these drugs as well as provide structural and biophysical understanding of their properties. We suggest that the designed nanocarriers can serve as programmable nanostructures for the future design of new generations of advanced nanocarriers with potential cancer- and patient-specific targeting properties.

## Graphical abstract

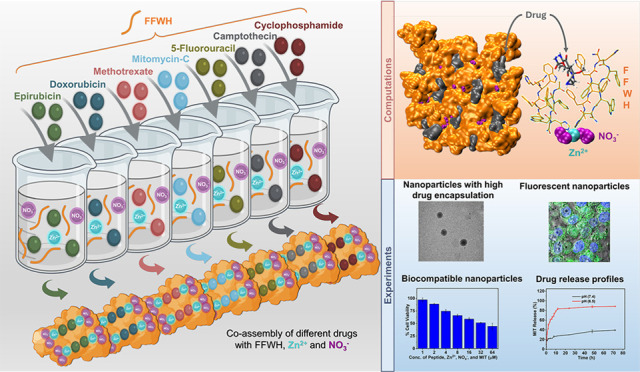

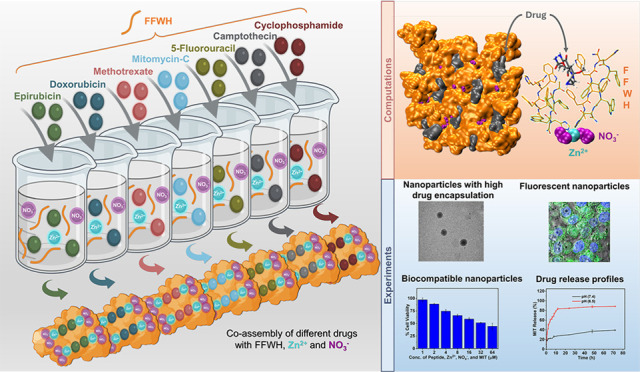

## Introduction

Self-assembling peptides represent a highly appealing class of nanomaterials with promising biomedicine applications, particularly in drug delivery. Nanomaterials comprising self-assembling and co-assembling peptide systems offer advantages, including potential biocompatibility and tunable bioactivity. They can be engineered for efficient targeting of specific sites, loading various drugs, and triggering drug release at disease locations. Hence, self-assembling and co-assembling peptide nanomaterials have been suggested as potential drug delivery systems for various applications, including cancer treatment, facilitating drug release and stability while minimizing side effects (reviewed in references).

Chemotherapy has been a standard treatment for primary and metastatic cancer for many years, but still its clinical effectiveness is often limited, especially when administered as a single-agent therapy (monotherapy).(18) Key challenges related to monotherapy consist of drug resistance at cellular and tumor levels, harsh tumor microenvironment that hinders drug penetration and efficacy, tumor heterogeneity, and dose-limiting toxicity.(18) Combination chemotherapy regimens with two or more traditional anticancer drugs have been used for decades in clinical practice to treat various types of cancer.(18) It is important that the different drug components are delivered to the correct location at the designated time.(18) However, the intrinsic differences in the physicochemical and pharmacokinetic properties of the different drugs make it challenging, unless an efficient drug carrier is used.(18) The strategy of codelivering combinations of drugs using the same carrier has been increasingly applied in recent years.(18) In the clinic, sequential administration, compared to simultaneous administration, is suggested as the standard cancer chemotherapy(18) because of the prominent adverse effects and absence of obvious benefits in overall survival observed in clinical trials for simultaneously administered combination chemotherapy, despite a higher tumor response rate.(18) One of the ways for sequential administration is for the drugs to be delivered via separate nanocarriers.(18) Several nanocarrier systems have been studied for delivering drug combinations, with a few advancing to clinical trial stages.(18)

A series of studies have reported the computational and experimental design of cancer drug delivery systems utilizing peptide self-assembly.(2) Among others, previous studies have considered the importance of combining structure stability and drug encapsulation, fluorescence, and minimalism, where minimalism refers to using a minimal building block that can form ordered structures with a desired functionality. Our previous work demonstrated that cyclo-dihistidine peptides (Cyclo-HH) can self-assemble into supramolecular nanostructures, with an enhanced fluorescence in the presence of Zn^2+^ and NO_3_^–^ ions and a capacity to co-assemble with cancer drug Epirubicin; this resulted in the formation of a nanocarrier capable to effectively deliver the drug into cancer cells while allowing *in situ* monitoring. Prompted by the importance of efficiently encapsulating multiple cancer drugs, we subsequently demonstrated that Cyclo-HH can additionally co-assemble with Doxorubicin and Methotrexate, in the presence of Zn^2+^ and NO_3_^–^, but not efficiently for other drugs.(1) These studies highlighted the key role of His-Zn^2+^ in fluorescence, and the role of both ions Zn^2+^ and NO_3_^–^ in mediating interactions between peptides and drugs. They also showed the importance of His-Zn^2+^ coordination for both enhanced stability at neutral conditions and less stability at acidic conditions, which are key properties for cancer drug delivery systems considering the variation in pH conditions outside and within a cancer cell.

In this study, we initially used computational approaches to design four-residue histidine-containing peptides with the capacity to encapsulate a diverse range of cancer drugs and coordinate with Zn^2+^ for enhanced fluorescence. The list of drugs considered included Epirubicin (EPI), Doxorubicin (DOX), Methotrexate (MTX), Mitomycin-C (MIT), 5-Fluorouracil (5FU), Camptothecin (CPT), and Cyclophosphamide (CP), all commonly used in monotherapies and/or combination therapies (AC: Adriamycin (or Doxorubicin) and Cyclophosphamide, CAF: Cyclophosphamide, Adriamycin, and Fluorouracil, CMF: Cyclophosphamide, Methotrexate, and Fluorouracil, FEC: Fluorouracil, Epirubicin, and Cyclophosphamide) for breast cancer according to National Cancer Institute.(26) Additionally, they have been tested in combinations in clinical trials. In tandem, computations and experiments yielded a set of top-designed peptides for all drugs, and focused on a single peptide, FFWH. This was identified as the top consensus peptide forming co-assembled nanocarriers with each drug, possessing high structural and energetic stability, and efficiently encapsulating all cancer drugs. Additionally, the nanocarriers combined remarkable biocompatibility, enhanced fluorescence, and uptake into the cytoplasm of HeLa cells according to fluorescence spectroscopy, cell viability assays, and live-cell imaging using HeLa cells.

## Methods

### Computational Methods

#### M1: Computational Investigation of Novel Minimalistic Peptide Scaffolds in Complex with Orange G

Starting from the experimentally resolved structure of an amyloid-forming peptide KLVFFA from amyloid beta in complex with Orange G,(36) we investigated truncated peptides for their capacity to represent novel minimalistic scaffolds. We studied a three-residue peptide (^3^VFF^5^), a four-residue peptide (^2^LVFF^5^), and a five-residue peptide (^1^KLVFF^5^) for their capacity to encapsulate Orange G and maintain the scaffold’s structural integrity in comparison to ^1^KLVFFA^6^, which served as a control. Upon modeling the minimalistic peptide scaffolds (SM1(A)), we performed short “screening-like” MD simulations (SM1(B)), which depicted, based on structural analysis (SM1(C)), that the four-residue peptide scaffold represented a system that sufficed the two criteria: capacity to encapsulate Orange G, and highly maintain its structural integrity (Figure S1). Hence, the four-residue (^2^LVFF^5^) peptide scaffold was selected as the basis for further design (Figure S2).

#### M2: Preparation of the Designable Four-Residue Peptide Scaffolds

The four-residue peptide scaffold (^2^LVFF^5^) was initially modified by introducing a histidine mutation at position four to facilitate Zn^2+^ coordination (SM2(A), Figure S3). Due to the different net charges of the cancer drugs considered for subsequent design, different scaffolds were prepared with different compositions of peptide termini and with varying ratios and placement of Zn^2+^ and NO_3_^–^ for charge neutrality in each system (SM2(A), Figure S4). The corresponding modified scaffolds were prepared (SM2(B), Figure S5) and simulated as detailed in Supporting Information (SM2(B)). The drugs under investigation were introduced by superimposing them onto Orange G for all modified scaffolds, respectively, resulting for every drug in seven Initially-Prepared Scaffolds (IPS_(1–7)_; corresponding nearly perfectly ordered idealistic assemblies) and in six Simulation-Extracted Scaffolds (SES_(1–6)_; corresponding to more realistic and compact but less ordered assemblies) (SM2(C), Figure S6). These were given as input to the design process.

#### M3: Evolution-Based Computational Design for All Designable Scaffolds

Starting from the LVFH peptide scaffolds, novel peptides were designed with the objective of possessing enhanced binding to different drugs while maintaining their ability to coordinate Zn^2+^. An in-house computational evolution-based design process was applied independently for all the drugs under investigation. The evolution-based design followed an iterative “Lock & Design as you Go” approach, where the best evaluated design per iteration was “locked” and given as input to the next iteration. Each iteration consisted of two stages: *Stage 1─Introduction of Mutations at Positions 1, 2, and 3*, based on evolution probabilities, and *Stage 2─Energy Evaluation* consisted of the association free energy of each drug molecule with the rest of the system. Details about the design process are presented in the Supporting Information (SM3(A), Figure S7). The “evolution-based computational design process” was performed for each drug independently, and our investigation focuses on nine *selected consensus peptides* (YWWH, FWWH, HWWH, WWWH, YYWH, LWWH, IWWH, QWWH and FFWH) based on two combined criteria: the drugs’ association free energy and the peptides’ aggregation propensity. A detailed explanation of the selection procedure is provided in sections SM3(B), SM3(C), and Results and Discussion.

#### M4: Computational Validation of Nine Selected Consensus Peptides─Simulating Ordered Assemblies with Different Drugs

We first used MD simulations to study whether the *selected consensus peptides* (YWWH, FWWH, HWWH, WWWH, YYWH, LWWH, IWWH, QWWH, and FFWH) co-assembled with drugs and ions have the capacity to maintain the integrity of the ordered assemblies they were designed based upon, originating from both IPS and SES scaffolds. We identified for each drug the IPS and SES corresponding systems with the lowest association free energy, and both were simulated (SM4(A)). Upon completion of simulations, corresponding to a total aggregated time of 12.6 μs across different peptides and drugs, structural and energetic analyses were performed (SM4(B)). The drugs’ consensus association free energy depicts a variation between WWWH, QWWH, FFWH, HWWH, YWWH and the rest (see Results and Discussion). Additionally, the early stage self-assembly properties of these were investigated (SM4(C)), depicting HWWH underperforming in its ability to form β-sheet-like configurations (see Results and Discussion). Peptides WWWH, QWWH, FFWH, and YWWH are further investigated computationally and experimentally and are referred to as *top consensus peptides*.

#### M5: Computational Investigation of the Top Consensus Peptides on the Early Stage Co-assembly with Different Drugs

At this stage, we investigated the early stage co-assembly properties of the *top consensus peptides*, WWWH, QWWH, FFWH, and YWWH with Zn^2+^ and NO_3_^–^ in the presence of drugs, in comparison to the absence of drugs, performed in M4. Each system was investigated for 2 μs. Both variations of termini were investigated for each peptide in the absence of drugs. In the simulations, we investigated multiple copies of each peptide in the presence of ions with multiple copies of the drugs. The copies of the peptides, drugs, and ions were initially placed in a random arrangement, maintaining the specific ratio determined during the design model per case of drug (SM5(A)). Upon the completion of simulations, corresponding to a total aggregated time of 72 μs across different peptides and drugs, we used in-house Fortran programs to identify multicomponent clusters comprising different components of peptides, ions, and drugs. Upon detection of the clusters, we extracted a series of structural properties (SM5(B)). FFWH outperformed the rest in its enhanced propensity to co-assemble into β-sheet-like configurations in the presence of drugs and ions, with the exception of EPI (see Results and Discussion), and thus, we extended the simulations of the particular peptide in the presence of drugs up to 4 μs, which allowed us to provide insights into the evolution of assembly, particularly on the peptides’ organization within the clusters and the formation of β-sheets. For the extended simulated time, we performed additional structural analysis of (SM5(C)). Finally, we calculated the association free energy of the drugs and peptides with the rest of the corresponding system as a function of the solvent accessible area within the most representative clusters per drug under investigation and in the corresponding ordered assemblies originating from IPS (SM5(D)).

##### Computational Tools Used

In summary, all simulations in this study were performed using explicit solvent in 95:5 IPA/DMF, with the exception of screening-like simulations in SM1(B) in which water was used as a solvent; additional ions were added accordingly to neutralize the systems under investigation. Specifically: (a) “Multicomponent Assembler” along with the “Solution Builder” input generators provided by CHARMM-GUI were used to setup the simulations; (b) OpenMM(41) to conduct the simulations; (c) CHARMM(38) input files based on “PDB-Reader & Manipulator” input generator from CHARMM-GUI which were modified accordingly for drugs’ sampling, mutations and energy minimization; (d) Vinardo scoring function(45) to calculate energies during the design process; (e) Autodock4Zn scoring function(46) to calculate association free energies upon completion of simulations; (f) Wordom to calculate structural properties (i.e., radius of gyration, SASA); (g) VMD(49) for molecular graphics images, and additional (h) in-house Fortran and Linux scripts. Additional information is provided in the Supporting Information.

### Experimental Methods

#### M6: Experimental Investigation of the Top Consensus Peptides

The *top consensus peptides* Ac-FFWH-COO^–^, Ac-QWWH-COO^–^, Ac-WWWH-COO^–^, Ac-YWWH-COO^–^, corresponding and designed for EPI and DOX, as well as Ac-FFWH-CONH_2_, Ac-QWWH-CONH_2_, Ac-WWWH-CONH_2_, and Ac-YWWH-CONH_2_ corresponding and designed for all drugs except EPI and DOX were purchased from DGpeptides Co., Ltd. (Wuhan, China). Zinc nitrate (Zn(NO_3_)_2_), dimethylformamide (DMF), dimethyl sulfoxide (DMSO), isopropyl alcohol (IPA), and ethanol (EtOH) were purchased from Sigma-Aldrich (Rehovot, Israel). All the drugs, such as EPI, DOX, MTX, MIT, 5FU, CPT, and CP were purchased from Sigma-Aldrich (Rehovot, Israel). All materials were used as received without any further purification. Highly pure deionized water was processed using a Millipore purification system (Darmstadt, Germany) with a minimum resistivity of 18.2 MΩ cm.

##### Co-assembly of the Top Peptides with Zn^2+^, NO_3_^–^ and Cancer Drugs

The co-assembly of all peptides with Zn^2+^, NO_3_^–^ and with or without cancer drugs (EPI, DOX, MTX, MIT, 5FU, CPT, and CP) were performed in 95% IPA/DMF as previously described. The co-assembly of the peptides was performed independently using drugs EPI, DOX, MTX, MIT, 5FU, CPT, and CP. In order to prepare a fresh stock solution of the peptides, it was necessary to dissolve it in 95% (v/v) IPA/DMF at a concentration of four mol each of the designed peptides, one mol of Zn(NO_3_)_2_ (except in case of the MTX), and two mol of each cancer drug in water bath sonication, after which it was incubated at 80 °C for three h, then allowed to cool to room temperature overnight. In the case of MTX, the peptide:drug:zinc ratio was (4:2:2). The resulting suspension was centrifuged for 30 min at 14,000 rpm to remove any non-encapsulated excess drugs, unbound ions (Zn^2+^/NO_3_^–^), or unreacted salts (Zn(NO_3_)_2_). Deionized water was then used to wash the precipitate three times. A solid powder was obtained by lyophilizing the materials.

##### UV–Vis Spectrophotometry

The UV–vis spectroscopy was used to determine the drug encapsulation for all of the designed peptides. A 1.0 cm path length quartz cuvette and an Agilent Cary 100 UV–vis spectrophotometer were utilized to investigate the co-assembly of all the designed peptides, with Zn^2+^, NO_3_^–^, both in the presence and absence of the cancer drugs. The drug encapsulation was determined by comparing the relative UV–vis absorption peak to the control (reference) sample. In addition, we studied the release kinetics of drugs from co-assembled nanostructures using UV–vis spectroscopy.

#### M7: Experimental Studies on the Top Consensus Peptide

The assays described below were performed only for the top peptide with the FFWH sequence.

##### Transmission Electron Microscopy (TEM)

The TEM was used only for the top peptide, FFWH, as defined based on the drug encapsulation results and the computational results about the propensity for β-sheet-like configurations. The co-assembled nanostructures of Ac-FFWH-COO^–^ and Ac-FFWH-CONH_2_ with Zn^2+^, NO_3_^–^, and cancer drugs were deposited onto a glow discharge copper grid (400 mesh) coated with a thin carbon film and allowed to adsorb for 2 min. The excess solution was then removed from the grid, and it was dried under a vacuum. TEM images were captured using a JEM-1400Plus electron microscope operating at 80 kV. Images were analyzed using ImageJ software. To ensure accuracy, triple measurements were conducted.

##### Drug Release Profiles

For the nanostructures composed of the peptides Ac-FFWH-COO^–^ and Ac-FFWH-CONH_2_ with Zn^2+^, and NO_3_^–^ in comparison to pristine drugs, an in vitro drug release profile analysis was conducted using dialysis in PBS buffer (pH 7.4) or acetate buffer (pH 6.5). Dialysis was carried out in an incubator shaker at 37 °C, assuming that drug release would begin at normal human body temperature (37 °C) in two different buffers (pH 7.4 or 6.5). Aliquots (200 μL) were taken at predetermined intervals from the release reservoir solution at various time points for characterization using UV–vis spectrophotometry. The released studies of the various cancer drugs (EPI, DOX, MTX, MIT, 5FU, CPT, and CP) were determined by measuring their characteristic absorption wavelengths of 482, 480, 372, 365, 266, 365, and 270 nm using a calibration curve prepared under similar conditions.

##### Cell Viability Analysis

For 3-(4,5-dimethylthiazolyl-2)-2, 5-diphenyltetrazolium (MTT) assay, 1 × 10^6^ HeLa cells/mL were cultured in 96-well tissue culture plates (200 μL per well) and allowed to adhere overnight at 37 °C. The co-assembled nanostructures of the peptides Ac-FFWH-COO^–^ and Ac-FFWH-CONH_2_, Zn^2+^, NO_3_^–^, with or without cancer drugs, were added to the cells with varying concentrations of 1–64 μM for 24 h. Only a medium without nanostructures was used as the negative control. The pristine drugs were also used as controls. Briefly, 20 μL of 5 mg/mL MTT reagent was dissolved in PBS and mixed with 180 μL of fresh medium. Further, this mixture (20 μL) was added to each of the 96 wells, followed by 4 h incubation at 37 °C. Afterward, the medium containing MTT was removed after 4 h, and 100 μL of extraction buffer (100% DMSO) was added to each well, followed by 30 min of incubation at 37 °C in the dark. Finally, absorbance intensity was measured using a multiplate reader at 575 nm.

##### Live Cell Imaging

Live cell imaging of HeLa cells incubated with co-assembled nanostructures using Ac-FFWH-COO^–^ and Ac-FFWH-CONH_2_ peptides was obtained using confocal microscopy. The pristine drugs were also taken as controls in order to compare the co-assembled nanostructures. Briefly, HeLa cells were grown in glass-bottom dishes until they reached 70 to 80% confluency. The cells were then cultured in media containing drug-*co*-assembled nanostructures at a concentration of 4 μM for 12 h. Following incubation, the cells were washed three times with 1x PBS and further stained with a dye diluted 1:1000 in PBS for 15 min at room temperature in the dark to stain the nuclei. The cells were then washed three times with PBS. Imaging was performed using an SP8 inverted confocal microscope (Leica Microsystems, Wetzlar, Germany). In confocal microscopy, the excitation and emission absorbance ranges were 488 and 510–590 nm, respectively, for EPI, DOX, MTX, MIT, 5FU, CPT, and CP.

## Results and Discussion

### Derivation of Novel Minimalistic Peptide Scaffolds and Their Use in Computational Design

Starting from the experimentally resolved structure of an amyloid-forming peptide KLVFFA from amyloid beta in complex with Orange G we investigated several possible three-, four-, five-, and six-residue peptides for their capacity to maintain a high degree of structural integrity in complex with Orange G, in our effort to derive a novel minimalistic peptide scaffold for subsequent design in this study (Figure S1). Our analysis deduced to the four-residue peptide LVFF (Figure S1 and S2), and subsequently LVFH, at which a histidine residue was introduced for Zn^2+^ coordination and enhanced fluorescence in place of phenylalanine at position four (Figure S3). Due to the different net charges of the cancer drugs considered for subsequent design, to obtain charge neutrality in each designed system, different scaffolds were prepared with different compositions of peptide termini and with varying ratios and placement of Zn^2+^ and NO_3_^–^ (Figure S4, S5).

**Figure 1:**
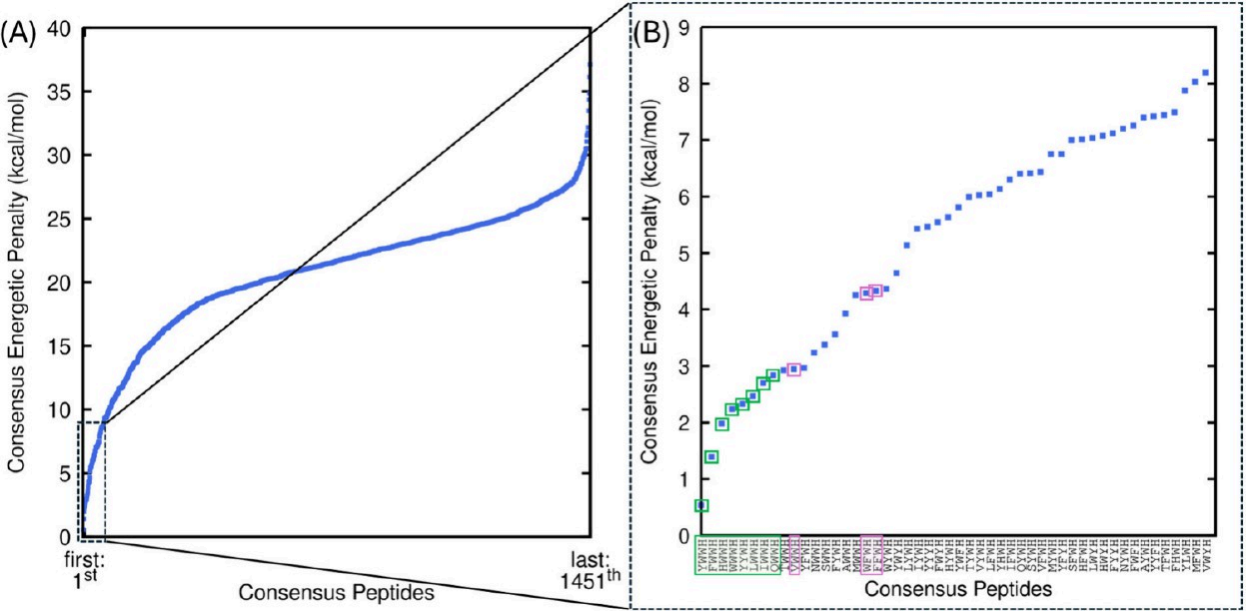
(A) Consensus Energy Penalty (kcal/mol) for all the 1451 consensus peptides identified throughout the evolution-based computational design process. (B) Consensus Energy Penalty (kcal/mol) of the top 50 ranked consensus peptides. The consensus peptides selected based on the drugs’ association free energy are highlighted in the green boxes (YWWH, FWWH, HWWH, WWWH, YYWH, LWWH, IWWH, QWWH) and the consensus peptides selected based on the aggregation propensity are highlighted in the purple boxes.

The scaffolds, representing either nearly perfectly ordered idealistic assemblies (referred to as “Initially Prepared Scaffolds”; IPS) or more realistic and compact but less ordered assemblies (referred to as “Simulation-Extracted Scaffolds”; SES), were provided as input to a computational design process (Figure S6). In summary, histidine was preserved at position four for Zn^2+^ coordination, and mutations were introduced at the first three residue positions. A series of innovative components were considered during the design process, the key components of which are provided as follows. Prior to introducing mutations, multiple drug orientations and positions were considered, aiming to identify the optimum designs for any drug binding mode, inspired by our lab’s previous studies. Mutations were introduced aiming at “evolutionary” improving the drug association free energy with the peptide materials, based on evolution probabilities.(54) In this “evolution-based computational design process”, mutations were not addressed sequentially, but rather they were introduced aiming to mimic how nature would potentially design peptide-materials encapsulating cancer drugs in a data free approach, i.e., without any “materialphore” models describing how amino acids bind to a drug. Designs with mutations leading to improved association free energy were locked, were used as a new starting point, and were unlocked only when a new improvement was identified. Importantly, the designs were constantly evaluated and were not evaluated at the end; rather, designs were constantly evaluated *in processu* (i.e., during the process), and importantly, they actively affected the process progression (Figure S7). Upon execution of multiple computational design runs per drug until convergence, designs which were common across different drugs were identified, similar to our previous study.(56) These designs were ranked based on a consensus-derived energetic penalty metric (Figure 1(A)), enabling the selection of the most promising designs from all generated common designs (Figure 1(B)). The corresponding methodology is provided in M1–M3, and the Supporting Information (SM1–SM3).

### Computational Validation of Nine Selected Consensus Peptides─Simulating Ordered Assemblies with Different Drugs

Upon performing the “evolution-based computational design process” for each drug independently, in what follows, our investigation focuses on nine *selected consensus peptides*, YWWH, FWWH, HWWH, WWWH, YYWH, LWWH, IWWH, QWWH and FFWH, based on two combined criteria: the drugs’ association free energy (Figure 1) and the peptides’ aggregation propensity (Figure S8). The top 20 peptides, ranked by the lowest consensus energetic penalty derived from the drugs’ association free energy during the design process, followed the pattern: hydrophobic/polar/aromatic in the first position, followed by an aromatic residue at position 2, and predominantly tryptophan (with one exception) at position 3 (Figure 1(B)). The following peptides with the lowest consensus energetic penalty ranking were selected and investigated on what follows: YWWH, FWWH, HWWH, WWWH, YYWH, LWWH, IWWH, and QWWH (Figure 1(B), see with green color). Also, from the list of top 20, the following remaining peptides, VWWH, WFWH, and FFWH (Figure 1(B), see with purple color), were further investigated due to their highest aggregation propensity compared to the remaining ones (WYWH, YFWH, FYWH, TWWH, MWWH, NWWH, YWYH, SWWH, and AWWH (Figure S8(A)). The short MD simulations of the three peptides VWWH, WFWH, and FFWH indicated that FFWH is more prone to aggregate and form β-sheet-like configurations compared to the rest (Figure S8(B–C)), thereby completing the set of nine *selected consensus peptides*. Hereafter, the term “consensus peptides” refers to peptides with identical sequences amidated N-terminal, but distinct C-terminal based on the target drug. Peptides for charged drugs EPI and DOX possess a −COO^–^ C-terminal, whereas all the other drugs possess a −CONH_2_ C-terminal; e.g., FFWH, when co-assembled with all drugs except EPI and DOX, it corresponds to Ac-FFWH-CONH_2_, and when co-assembled with EPI and DOX, it corresponds to Ac-FFWH-COO^–^. The peptides’ termini as well as the peptides:drugs:Zn^2+^ ratio were computationally determined for each drug system to ensure system neutrality for enhanced co-assembly. Considering the different termini and the charge of each drug, the peptides:drugs:Zn^2+^ ion ratio was adjusted to ensure neutrality of the systems. Thus, in what follows, systems comprising EPI, DOX, MIT, 5FU, CPT, and CP follow a ratio of [4]:[2]:[1], while the systems comprising MTX follow a ratio of [4]:[2]:[2]. These design considerations on the peptides’ termini and the peptides:drugs:Zn^2+^ ions ratio were applied to the computationally simulated systems and the experimental co-assembled nanoparticles.

As part of the computational validation, simulations of the ordered assemblies were performed to investigate the drug nanocarriers’ structural and energetic stability as well as the nanocarriers’ capacity to efficiently encapsulate cancer drugs. The corresponding methodology is provided in M4, and Supporting Information (SM4).

Within the simulations, the peptides’ stability was verified, while a nearly perfect drug encapsulation of the charged drugs, EPI, DOX and MTX, with variations across the neutral drugs was observed (Figure S9). Nevertheless, the drugs’ consensus association free energy shows a variation between WWWH, QWWH, FFWH, HWWH and YWWH and the rest, with HWWH underperforming in its ability to form β-sheet-like configurations (Figure S10). Peptides WWWH, QWWH, FFWH, and YWWH are further investigated computationally and experimentally and are referred to as *top consensus peptides*.

### Computational Investigation of the Top Consensus Peptides─Simulating Early Stage Co-assembly with Different Drugs

The early stage co-assembly properties of the *top consensus* peptides were additionally investigated independently for each drug under investigation using simulations, where multiple copies of the different components, peptides, drugs, and ions were placed initially in a random arrangement. Upon the completion of 2 μs of the simulations, in-house Fortran programs detected the formation of multicomponent co-assembled clusters of different sizes, across all the systems under investigation, including each combination of the *top consensus* peptides with each drug (Figure S11). A structural analysis of the formed clusters revealed that all of the *top consensus* peptides presented nearly identical tendencies to encapsulate the different drugs under investigation. Particularly, the percentage of the encapsulated drug molecules out of all the available drug molecules increased with cluster size across all simulated systems, independent of peptide sequence or drug type (Figure S12). Structural analysis further revealed that the composition of the formed clusters reflected the designed component ratios for each drug across all of the peptides. Notably, the designed peptide:drug:Zn^2+^ ratio was approximately 4:2:1 for systems with EPI, DOX, MIT, 5FU, CPT, and CP, and 4:2:2 for systems with MTX (Figure S13). Interestingly, this proportional consistency in component ratios was observed regardless of the cluster sizes and universally across all systems, which suggested that all *top consensus peptides* have a similar propensity to co-assemble with the different drugs in the presence of the respective ions. Hence, all *top consensus peptides* WWWH, QWWH, FFWH and YWWH exhibited a high tendency to co-assemble, encompassing notable drug and Zn^2+^ encapsulation. However, FFWH appeared to possess an enhanced propensity to co-assemble into β-sheet-like configurations compared to the rest of the peptides in the presence of drugs and ions (with the exception of EPI) (Figure S14). Notably, as shown below, in extended simulations, this tendency increases with time, as expected. Similar simulations were performed complementary for the *top consensus* peptides in the absence of drugs and the structural analysis showed that FFWH still acquired a higher propensity to form β-sheet-like configurations compared to the rest pristine peptides (Figure S14). The corresponding methodology is provided in M5, and the Supporting Information (SM5).

### Experimental Investigation of the Top Consensus Peptides’ Drug Encapsulation Properties

The *top consensus* peptides were additionally investigated experimentally for their ability to co-assemble with each drug under investigation, in the presence of Zn^2+^ and NO_3_^–^. To allow their co-assembly, each peptide was mixed under controlled experimental conditions with each drug in the presence of Zn^2+^ and NO_3_^–^ under the computationally designed ratios, resulting in nanoparticle formation across all the systems under investigation. The drug encapsulation in the formed nanoparticles was further assessed using UV–vis spectroscopy at the corresponding wavelengths for each drug (EPI: 482 nm, DOX: 480 nm, MTX: 372 nm, MIT: 365 nm, 5FU: 266 nm, CPT: 365 nm, and CP: 270 nm). The *top consensus* peptides presented high drug encapsulation across all the neutral drugs, MIT, 5FU, CPT and CP, ranging between 86% and 97%. The encapsulation of MTX was also high (>93%) for all the consensus peptides besides YWWH (∼52%). Similarly, DOX encapsulation was very high for FFWH and WWWH (>93%) but reduced (∼50%) for QWWH and YWWH. Interestingly, the EPI encapsulation across all the consensus peptides was significantly lower compared to the other drugs (<36%), with WWWH showing the lowest efficiency (∼23%) (Figure S15). The lowest percentage of EPI being encapsulated by all the peptides compared to its epimer DOX could be potentially attributed to its p*K*_a_ value,(57) which is lower than DOX. Hence, the experimental results on drugs’ encapsulation combined with the aforementioned computational results conform to selecting FFWH as the top peptide for further computational and experimental investigation. The corresponding methodology is provided in M6.

### Computational Structural Investigation of FFWH─Extending Simulations of Its Early Stage Co-assembly with Different Drugs

**Figure 2:**
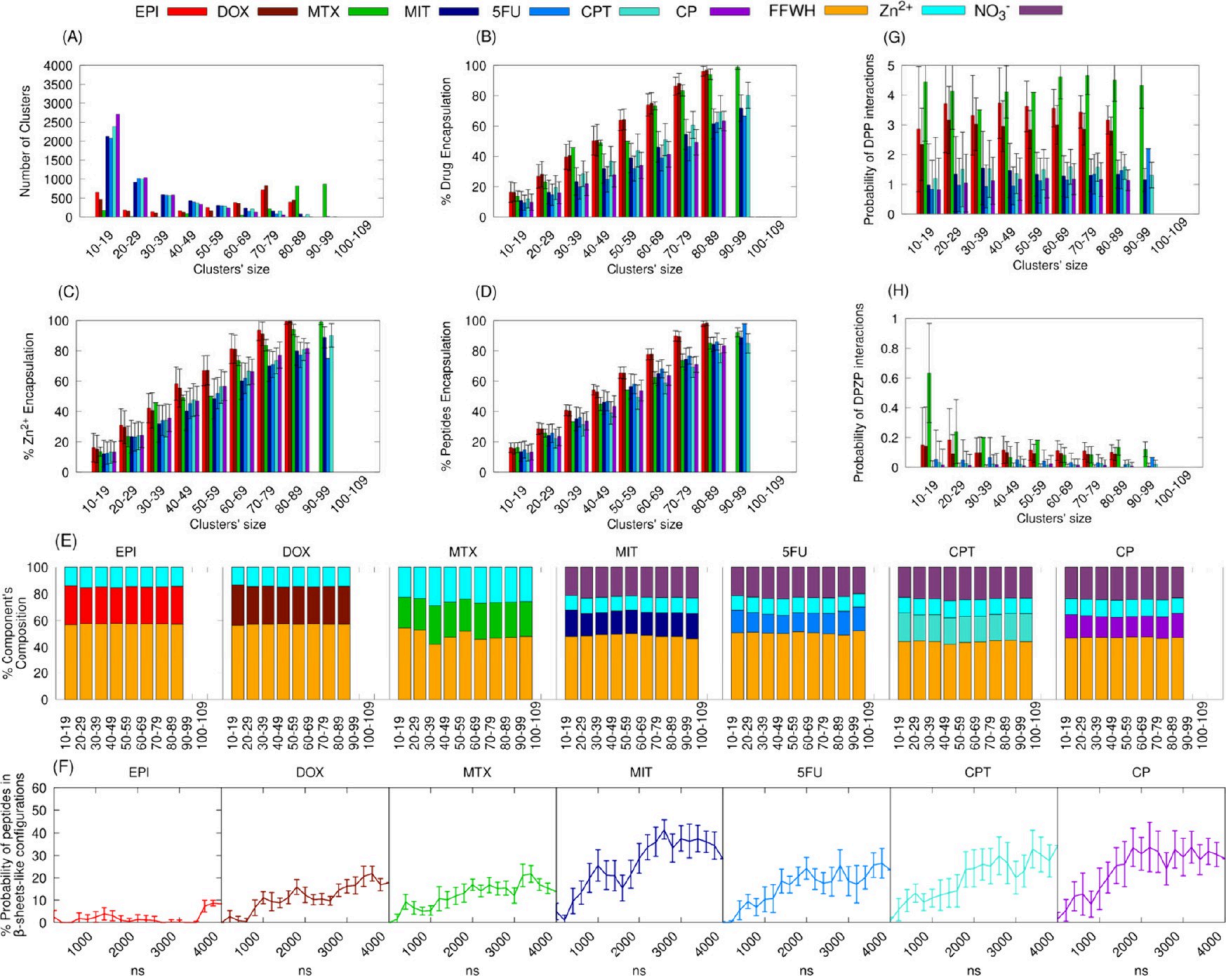
(A) Number of clusters as a function of clusters’ size. (B) Percent of drug encapsulation as a function of clusters’ size. (C) Percent of Zn^2+^ encapsulation as a function of clusters’ size. (D) Percent of peptides encapsulation as a function of clusters’ size. (E) Percent of components’ composition as a function of clusters’ size for clusters with EPI (red), DOX (maroon), MTX (green), MIT (dark blue), 5FU (light blue), CPT (purple) and CP (light purple). (F) Percent probability of peptides in β-sheet-like configurations as a function of time for clusters with EPI (red), EPI (red), DOX (maroon), MTX (green), MIT (dark blue), 5FU (light blue), CPT (purple) and CP (light purple). (G) Probability of a drug molecule to mediate two peptides as a function of clusters’ size. (H) Probability of a drug molecule to mediate two peptides and one Zn^2+^ simultaneously as a function of the clusters’ size for clusters with EPI (red), DOX (maroon), MTX (green), MIT (dark blue), 5FU (light blue), CPT (purple) and CP (light purple).

Simulations investigating the early stage co-assembly of FFWH with different cancer drugs were extended up to 4 μs at this stage. In particular, we observed that the formation of clusters with a large number of entities begins at early stages and extends up to the end of the simulations (EPI, DOX, MTX; Figure S16(A–C)), while for other systems, extension of the simulations enabled us to observe convergence (MIT, 5FU, CPT; Figure S16(D–F)), with the only exception (CP; Figure S16(G)) and which a sudden increase is observed in the last time window (3.8–4.0 μs). Hence, in the extended simulations, analysis revealed a higher tendency for cluster formation in simulated systems containing EPI, DOX, or MTX compared to that in those with neutral drugs (Figure 2(A)). The percentage of the drugs encapsulated increased as a function of the clusters’ size for all the drugs, but for EPI, DOX and MTX it was consistently higher compared to the rest of the drugs. The percentage of EPI, DOX and MTX encapsulated in the largest co-assembled clusters per case was very high, ∼96%, ∼97%, ∼99%, whereas for the MIT, 5FU, CPT, CP was ∼72%, ∼67%, ∼80% and ∼63% respectively (Figure 2(B)). Similar tendency across the clusters co-assembled by the different drugs was observed also for the Zn^2+^ and peptides encapsulation, which were increased as a function of the clusters’ size with these percentages in the clusters co-assembled by EPI, DOX and MTX being consistently higher than the rest (Figure 2(C), 2(D)). Particularly, the percentage of Zn^2+^ encapsulated in the largest clusters co-assembled by EPI, DOX or MTX was ∼99%, whereas for the MIT, 5FU, CPT, and CP was ∼89%, ∼77%, ∼90% and 82%, respectively (Figure 2(C)). Likewise, the percentage of peptides encapsulated in the largest clusters co-assembled by EPI, DOX or MTX was ∼98%, ∼98% and ∼92%, while it was slightly lower for MIT, 5FU, CPT, CP, which was ∼89%, ∼98%, ∼85% and 83% respectively (Figure 2(D)). The components’ composition in the co-assembled clusters showed that, irrespective of the cluster’s size, the component ratio is stable and reminiscent of the designed component ratios for each drug (Figure 2(E)). This aligns with the observed increase in partial component (peptides, drugs, and Zn^2+^) encapsulation as cluster size grows. Additionally, the probability of the peptides that co-assemble in β-sheet-like configurations increases as a function of time, as anticipated, with the highest values observed for the neutral drugs, followed by DOX and then MTX (Figure 2(F)). Overall, neutral drugs showed a higher tendency for FFWH to form β-sheet-like configurations. Additionally, structural analysis revealed that the charged drugs EPI, DOX, and MTX exhibited a higher propensity to mediate interactions between two peptides, as well as between two peptides and Zn^2+^ simultaneously, compared to the neutral drugs (Figure 2(G), (H)). This suggests that even during the early stages of FFWH co-assembly with each drug, units with a portion of features reminiscent of those in the designed structures can be formed. Representative clusters formed by the co-assembly of the FFWH peptide with Zn^2+^, NO_3_^–^, and each one of the drugs under investigation were selected and presented in Figure S17. The corresponding methodology is provided in M5, and Supporting Information (SM5).

### Experimental Studies on the Morphological and Structural Properties of Nanoparticles Formed by FFWH

**Figure 3:**
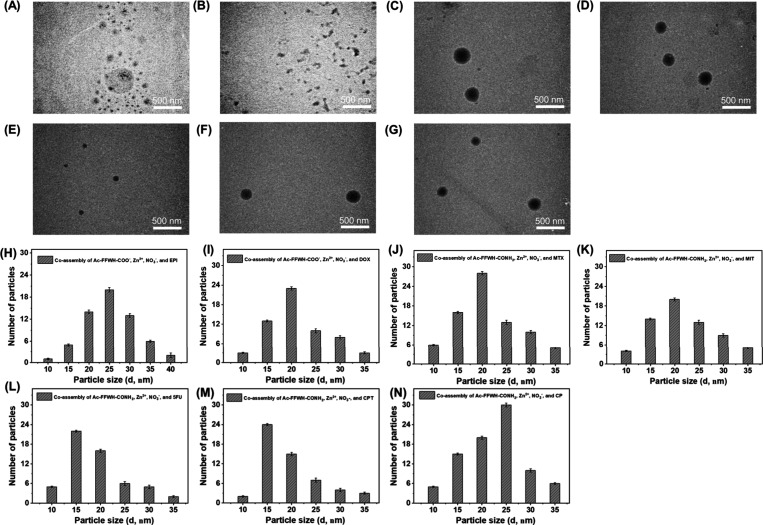
TEM images of the nanoparticles formed by the co-assembly of Ac-FFWH-COO^–^, Zn^2+^, and NO_3_^–^ with (A) EPI, and (B) DOX. TEM images of the nanoparticles obtained by the co-assembly of Ac-FFWH-COONH_2_, Zn^2+^, and NO_3_^–^ with various cancer drugs (C) MTX, (D) MIT, (E) 5FU, (F) CPT, and (G) CP. The images on the bottom (H–N) show a particle size distribution based on the TEM images.

After the drug encapsulation studies were successfully optimized, TEM analysis was carried out to verify the morphological properties of the formed nanostructures comprising the FFWH peptide. According to the TEM images, the average diameters of the nanoparticles formed by the co-assembly of the FFWH peptide, Zn^2+^, NO_3_^–^, and EPI, DOX, MTX, MIT, 5FU, CPT, or CP were ∼23, ∼18, ∼25, ∼23, ∼21, ∼24, and ∼28 nm, respectively (Figure 3). The variation in the nanoparticle sizes might be the result of the different percentages of the drugs being encapsulated, combined with the different sizes of the drugs, demonstrating that the presence of the drugs during the co-assembly could influence the nanostructures’ diameter. Overall, all the nanoparticles had a diameter higher than 10 nm and smaller than 100 nm, which is generally considered as a suitable range of nanoparticles for cancer therapy, as nanoparticles of this size can effectively deliver drugs and achieve enhanced permeability and retention effect.(58) The corresponding methodology is provided in section M7.

### Assessing Energetic Stability of Drugs and Peptides within Simulations Investigating Early Stage Co-assembly and Ordered Assemblies of FFWH

**Figure 4:**
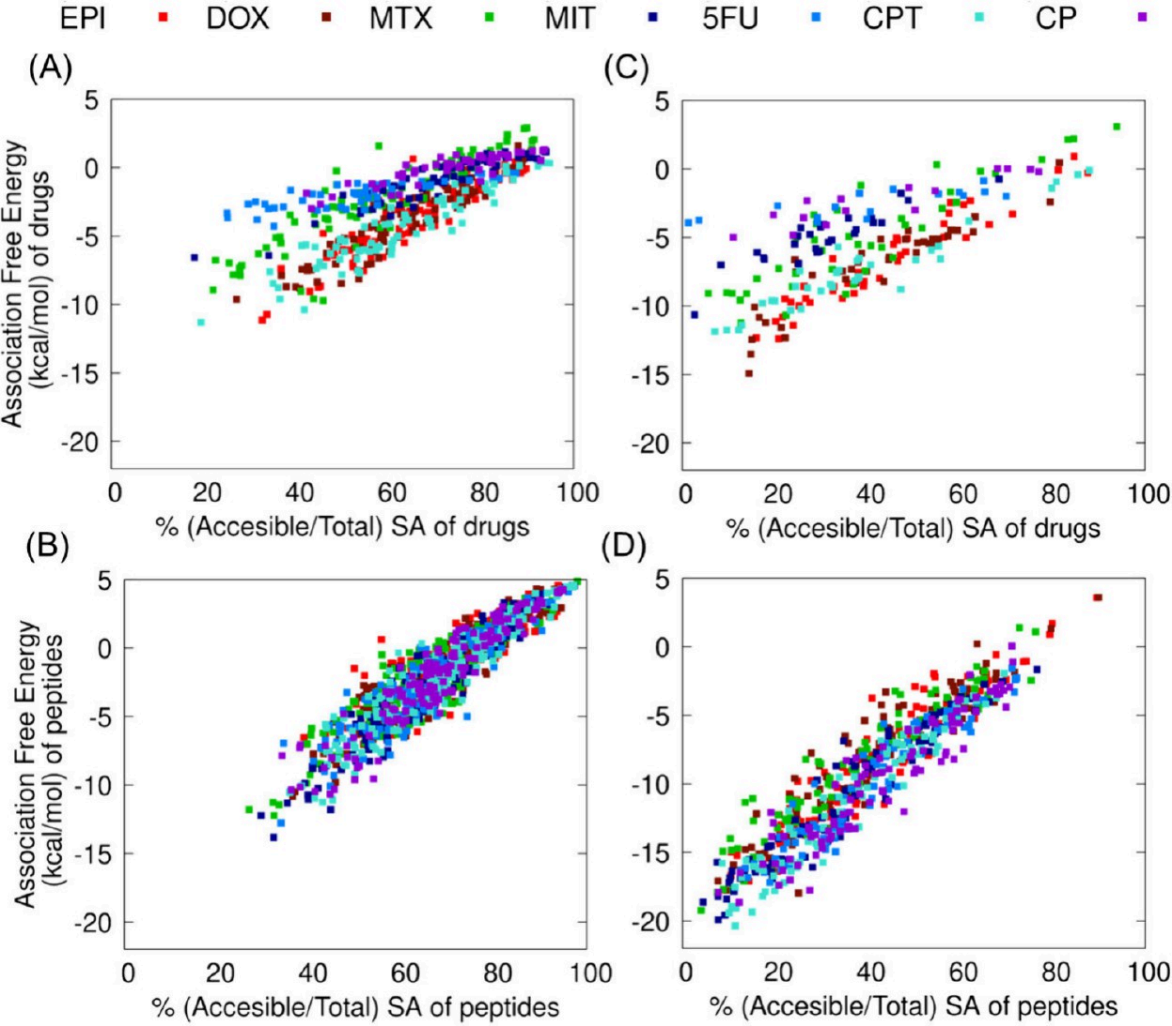
Association-free energy (kcal/mol) of drugs (A, C) and FFWH peptides (B, D), to the rest of the system, plotted as a function of the percentage of solvent accessible surface area divided by the total surface area of each drug or peptide. The calculations correspond to representative clusters formed in the simulations of the early stage co-assembly (A, B) and in the ordered assemblies (C, D), respectively. The calculations were performed for systems comprising different drugs: EPI (red), DOX (maroon), MTX (green), MIT (dark blue), 5FU (light blue), CPT (turquoise) and CP (light purple).

To assess drugs and peptides’ energetic stability within the simulations, we computationally calculated the association free energy of every molecule (drug and peptide), independently, within representative clusters during the early stages of co-assembly, and within ordered assemblies. Explanation of how representative clusters were extracted from simulations investigating early stages of co-assembly is provided in the Supporting Information (SM5). Additionally, calculations for ordered assemblies were performed for the last snapshot of the simulations investigating ordered assemblies, as part of the computational validation provided above; additional details are provided in the Supporting Information (SM5). The results are presented as a function of the ratio of the solvent accessible over the total accessible surface area per molecule (Figure 4) in the simulation snapshot. For both drugs and peptides, the degree of burial correlated with lower association free energy to the rest of the system, irrespective of the different drug systems under investigation in both early stage co-assembly (Figure 4A, 4B) and in ordered assemblies (Figure 4C, 4D). Interestingly, comparison of the association free energies of the different drugs under investigation against the same degree of exposure showed that EPI, DOX, MTX, MIT and CPT exhibited lower association free energy compared to 5FU and CP in both early stage co-assembly (Figure 4A) and in the ordered assemblies (Figure 4C). However, within the early stage co-assembly, the association free energy of the drugs compared to the association free energy of peptides is rather comparable (Figure 4A, 4B), whereas in the ordered assemblies the association free energy of the peptides is significantly lower compared to that of the drugs (Figure 4C, 4D). This can be attributed to the increased number of peptides in β-sheet-like configurations in the ordered assemblies. These energy findings suggest that the drug interactions with the system not only do not disrupt the peptide interactions with the system but they can favorably coexist with the peptides’ β-sheet-like configurations. Variations in the drugs’ binding energies may correlate with the experimentally observed drug release behavior discussed below. While FFWH appears to be an optimally designed peptide for the seven drugs, differences in the drugs’ association free energies are anticipated due to their different physicochemical properties.

### Drug-Peptide Interactions within “Optimal” Pockets Observed in Simulations of FFWH Ordered Assemblies

**Figure 5:**
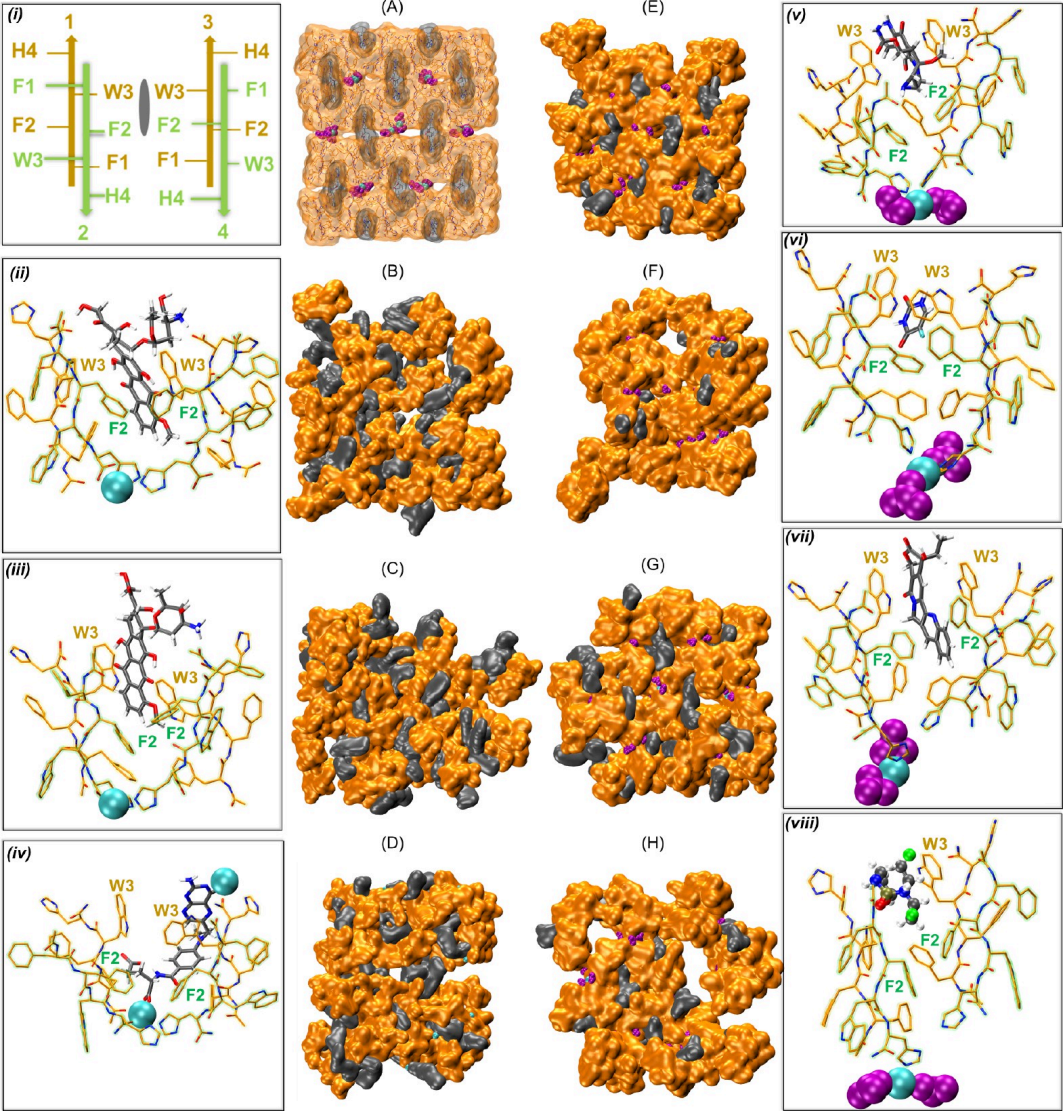
(A) Representation of the modeled ordered assemblies prior to simulations for a representative drug (CPT) along with (i) a panel showing a 2D graphical representation of the structural unit comprising one drug and two pairs of peptides in antiparallel β-sheets (peptide 1–peptide 2 and peptide 3–peptide 4) with the two opposite pairs parallel orientated to each other (peptides 1 and 3 (orange) in back, peptides 2 and 4 (green) in front). Representation of the ordered assemblies of different systems with different drugs (B) EPI, (C) DOX, (D) MTX, (E) MIT, (F) 5FU, (G) CPT, and (H) CP. Panels (ii–viii) show a representative structural unit extracted from the ordered assemblies from (B–G), respectively. In (A–H), FFWH peptides are shown in orange quick surface representation, the drugs in gray VDW representation, Zn^2+^ in cyan vdW representation, and NO_3_^–^ in purple VDW representation, while in (A) only, peptides and drugs are shown in a transparent representation and their heavy atoms are shown in licorice. In (ii–viii), peptides are shown with orange licorice and drugs with gray licorice, while additionally, to discriminate between peptides on the front and peptides on the back, peptides on the front layer are shown with a green outline, while the peptides on the back layer are shown with a yellow outline. Molecular graphics images were created using VMD.(49)

The high degree of encapsulation within the simulations of the ordered assemblies enabled us to provide insights into how each drug can optimally bind to the designed peptide systems. The FFWH ordered assemblies with each drug that are visually inspected below are the same as the ones used for the energy calculations in the section above. Visual representation of these ordered assemblies is shown in Figure 5(A–H). We identified and visually inspected the “optimal” pocket per drug, defined as the one with the lowest binding free energy drug under the condition that the two pairs of peptides enclosing the drug were in ordered β-sheets. Particularly, each pocket includes one drug and two pairs of peptides in antiparallel β-sheets (pair 1: peptides 1–2 and pair 2: peptides 3–4, Figure 5A, 5i) with the two opposite pairs parallel orientated to each other (peptides 1 and 3 (orange) in the back, peptides 2 and 4 (green) in the front, Figure 5i). Based on this configuration, residues F2 and H4 from the front-layer peptide pair (green), along with F1 and W3 from the back-layer peptide pair (orange), contribute mainly to drug binding (Figure 5i).

For the case of EPI (Figure 5B), interactions include a parallel π–π stacking between the aromatic rings of EPI and the W3 indole ring (orange right, Figure 5ii), as well as parallel or T-shaped π–π stacking with the F2 benzyl rings (green left and right, Figure 5ii). Besides the hydrophobic interactions, hydrogen bonds are formed between the hydroxyl group of EPI and the carboxyl peptide termini (orange, left, Figure 5ii) and a salt bridge is formed between the positively charged group of EPI and the carboxyl peptide termini (orange, right, Figure 5ii). Similarly, in DOX (Figure 5C), hydrophobic interactions occur between the methyl group of DOX and F2 (green, right, Figure 5iii) as well as a hydrogen bond between the charged group of DOX and the acetylated N-termini (orange, right, Figure 5iii). DOX is sandwiched between the two W3 indole rings (orange left and right, Figure 5iii). Within the MTX ordered assembly (Figure 5D), hydrophobic interactions are formed between the CH- groups of MTX and F2 benzyl rings (green left and right, Figure 5iv), while aromatic interactions occur between the benzyl ring of MTX and W3 indole ring (orange right, Figure 5iv), which also has the indole nitrogen hydrogen bonded with the nitrogen-including rings of MTX. Hydrogen bonds are also formed between the polar groups of MTX with some of the backbone atoms (orange left, Figure 5iv), while additionally, the negatively charged group of MTX is coordinated with the Zn^2+^ in the histidine pocket. Within the MIT ordered assembly (Figure 5E), the aromatic ring of MIT is sandwiched between the two W3 indole rings (orange right and left, Figure 5v). Additionally, hydrophobic interactions occur between the CH-groups of MIT and F2 (green right, Figure 5v), while the polar groups of MIT form hydrogen bonds with the indole nitrogen of W3 (orange left, Figure 5v), as well as with the acetylated and amidated termini (orange left and green left, Figure 5v). In the case of 5FU (Figure 5F), an aromatic cage is formed by the two W3 residues and a single F2 residue (orange left and right, green left, Figure 5vi), creating a binding site for 5FU. Regarding the CPT case (Figure 5G), the drug is sandwiched between the W3 indole rings (orange right and left, Figure 5vii), while parallel and T-shaped π–π stacking occurs between the aromatic group of CPT and the F2 rings (green left and right, Figure 5vii). Additionally, a hydrogen bond exists between the hydroxyl group of CPT and the W3 indole nitrogen (orange left, Figure 5vii). For the case of CP (Figure 5H), aromatic interactions are formed by the aromatic part of the drug and the W3 indole ring (orange right, Figure 5viii), while hydrophobic interactions are formed between the CH– groups of CPT and the F2 ring (green right, Figure 5viii). Additionally, the polar groups of drugs are hydrogen-bonded to peptides’ backbone atoms (orange left, Figure 5viii).

Notably, upon further investigation of the ordered assemblies in the final simulation snapshots for different drugs, all Zn^2+^ (100%) remained within the ordered assemblies for all drugs under investigation. For all neutral drugs, all Zn^2+^ were coordinated with the deprotonated nitrogen of at least a histidine,(59) and the corresponding percentage for MTX was 90% of Zn^2+^. For DOX and EPI, this dropped to 35.72% and 14.3%, respectively, due to the fact that Zn^2+^ were predominantly coordinating with the negatively charged carboxylic C-termini, but were still in the proximity of the histidine imidazole ring; notably, the peptides’ C-terminal was amidated-neutral in all other drugs. Interestingly, but not surprisingly, peptides maintained a high-quality to excellent β-sheet formation. Particularly, peptides were maintained in β-sheet-like conformations (according to extended or β-bridge definitions and STRIDE calculations in VMD(49)) in neutral drugs (100% for MIT, 5FU, CPT and 99% for CP), while the percentage of peptides in β-sheets is somewhat lower (∼82%) for EPI, DOX, and lower (∼66%) for MTX. The latter could be attributed to the fact that while MTX structures remained in a cluster, its larger size could partly disfavor β-sheet interactions. These results are also in line with the fact that neutral drugs better promote β-sheet interactions in simulations investigating the early stage co-assembly properties of the systems.

### Drug Release Profiles of FFWH Drug Nanoparticles

**Figure 6:**
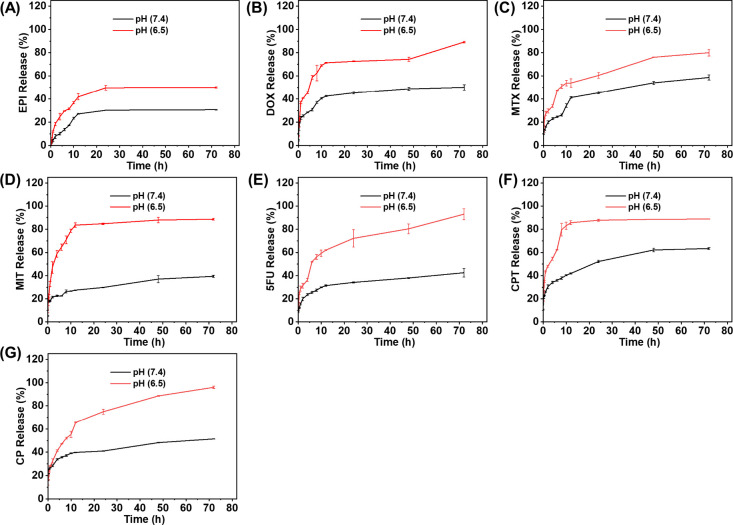
Drug release profiles of nanostructures that were formed by the co-assembly of Ac-FFWH-COO^–^, Zn^2+^, and NO_3_^–^ with (A) EPI and (B) DOX. Drug release profiles of nanostructures obtained by the co-assembly of Ac-FFWH-CONH_2_, Zn^2+^, and NO_3_^–^ with (C) MTX, (D) MIT, (E) 5FU, (F) CPT, and (G) CP in 3.5 kDa dialysis chambers at two different pH values (pH 6.5 or 7.4).

The UV–vis absorption spectroscopy was used to evaluate the release profiles of each drug encapsulated in the formed nanoparticles at different intervals (from 0 to 72 h) (Figure 6). Overall, all drugs exhibited similar release profiles in both pH conditions, with an initial rapid release followed by a plateau. Drug release was consistently higher in acidic conditions, showing that the release of all drugs was facilitated under the acidic conditions, similar to the cancer cells’ environment (red line higher than the black line at any time interval). Those types of drug release profiles show nearly constant drug release rate, which can lead to a controlled therapeutic effect, ideal for cancer drug release.(60) Focusing on the initial phase of rapid drug release (∼10–12 h), before the plateau began, it was observed that the release rate differences between pH conditions varied among drugs. For EPI, DOX, and MTX, the release rates (slope) at pH 6.5 and 7.4 were similar, whereas for the other drugs, the release was significantly higher under acidic conditions. After the initial phase of the rapid release, the difference in the drug release between pH 6.5 and 7.4 was less pronounced for EPI, DOX, and MTX compared with the other drugs. This suggests that the formed nanocarriers, including EPI, DOX and MTX, might be less pH sensitive. This could potentially be interpreted by stronger drug-carrier interactions, which is in line with lower association free energies according to computational studies (Figure 4). After 72 h, the release of drugs from the co-assembled nanostructures varied between pH conditions. At pH 7.4 vs pH 6.5, EPI showed 30.75% vs 50% release, DOX 50.10% vs 89.21%, MTX 58.66% vs 80.06%, MIT 43.83% vs 88.66%, 5-FU 42.53% vs 92.99%, CPT 63.39% vs 88.78%, and CP 51.46% vs 96.00% (Figure 6). Interestingly, after 72 h, the nanoparticles encapsulating 5FU and CPT exhibited nearly complete drug release, compared to the rest of the drugs. This could be possibly related to the higher computationally calculated association free energy of these drugs in their more deeply buried states within the nanocarriers compared to the other drugs under investigation.

### Cytocompatibility Analysis of FFWH Drug Nanoparticles

**Figure 7:**
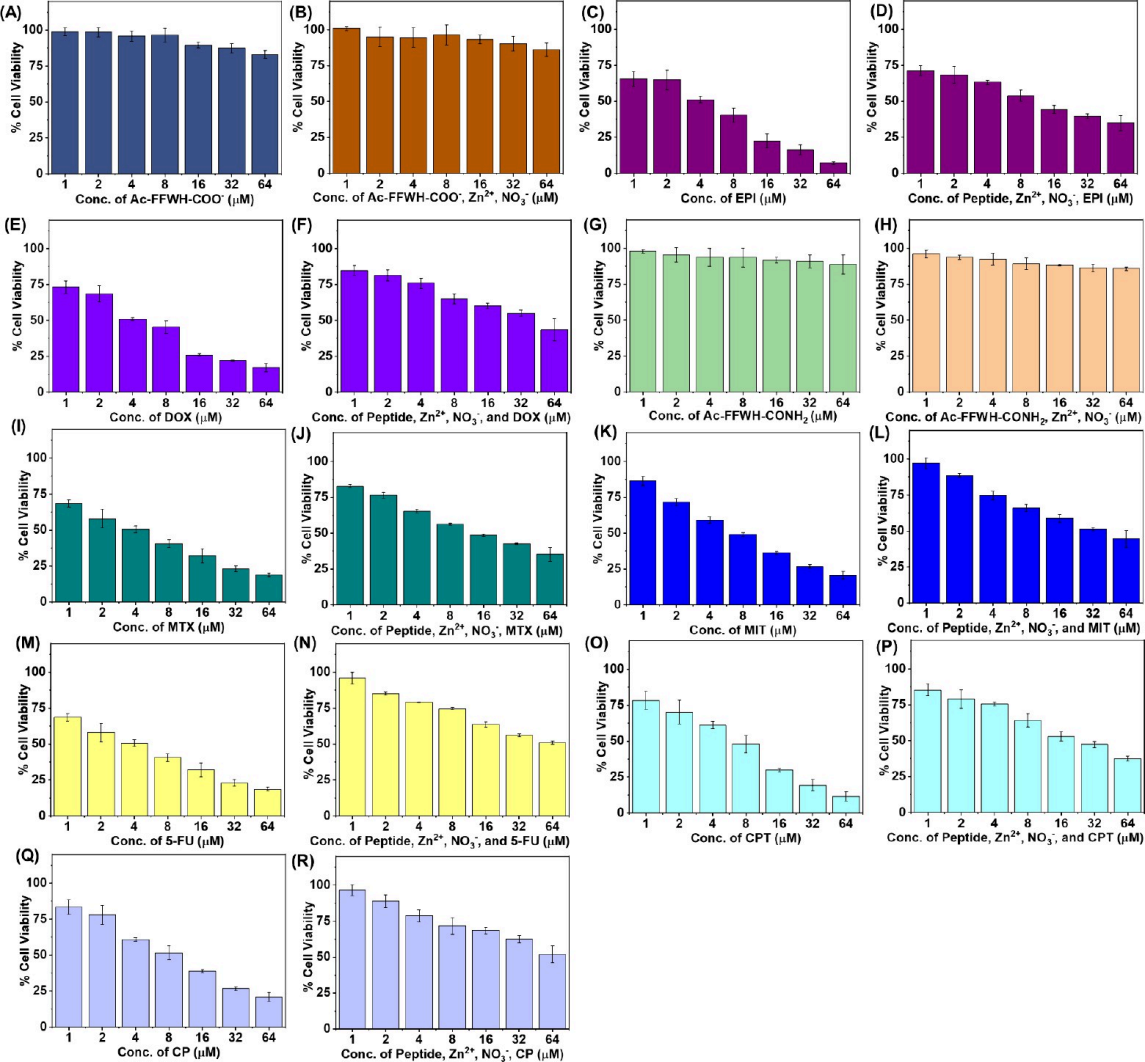
In vitro cell viability was assessed by MTT assays after incubation with (A) pristine Ac-FFWH-COO^–^, (B) co-assembled nanostructures of Ac-FFWH-COO^–^, Zn^2+^, and NO_3_^–^, (C) pristine EPI, (D) co-assembled nanostructures of Ac-FFWH-COO^–^, Zn^2+^, and NO_3_^–^ with EPI, (E) pristine DOX, (F) co-assembled nanostructures of Ac-FFWH-COO^–^, Zn^2+^, NO_3_^–^ with DOX, (G) pristine Ac-FFWH-CONH_2_, (H) co-assembled nanostructures of Ac-FFWH-CONH_2_, Zn^2+^, and NO_3_^–^, (I) pristine MTX, (J) co-assembled nanostructures of Ac-FFWH-CONH_2_, Zn^2+^, and NO_3_^–^ with MTX, (K) pristine MIT, (L) co-assembled nanostructures of Ac-FFWH-CONH_2_, Zn^2+^, and NO_3_^–^ with MIT, (M) pristine 5FU, (N) co-assembled nanostructures of Ac-FFWH-CONH_2_, Zn^2+^, and NO_3_^–^ with 5FU, (O) pristine CPT, (P) co-assembled nanostructures of Ac-FFWH-CONH_2_, Zn^2+^, and NO_3_^–^ with CPT, (Q) pristine CP, (R) co-assembled nanostructures of Ac-FFWH-CONH_2_, Zn^2+^, and NO_3_^–^ with CP, onto HeLa cells.

Following, we investigated the cytotoxicity of the co-assembled nanoparticles formed by the FFWH peptides with each one of the drugs on HeLa cells after 24 h of treatment at concentrations up to 64 μM. First, the nanoparticles formed by the FFWH peptides (Ac-FFWH-COO^–^ and Ac-FFWH-CONH_2_) demonstrated excellent biocompatibility both in the presence and absence of Zn^2+^, and NO_3_^–^, with the cell’s viability remaining ≥ 83% after 24 h of treatment at all the different concentrations under investigation (Figure 7A–B, 7G–H). Furthermore, the in vitro cell viability assays revealed that the co-assembled nanoparticles with each drug exhibited lower toxicity compared to the pristine drugs at the same concentrations (Figure 7C–D, 7E–F, 7I–J, 7K–L, 7M–N, 7O–P, 7Q–R), which could be related to the different amounts of drugs which were encapsulated and released from the co-assembled nanoparticles per case. Additionally, cell viability decreased with increasing treatment concentration for both the co-assembled nanoparticles and the pristine drugs, showing that the carriers do not interfere with the dose-dependent cytotoxic effect of the drugs, even if they enhanced the biocompatibility.

### Cellular Uptake Studies of FFWH Drug Nanoparticles into HeLa Cells

**Figure 8:**
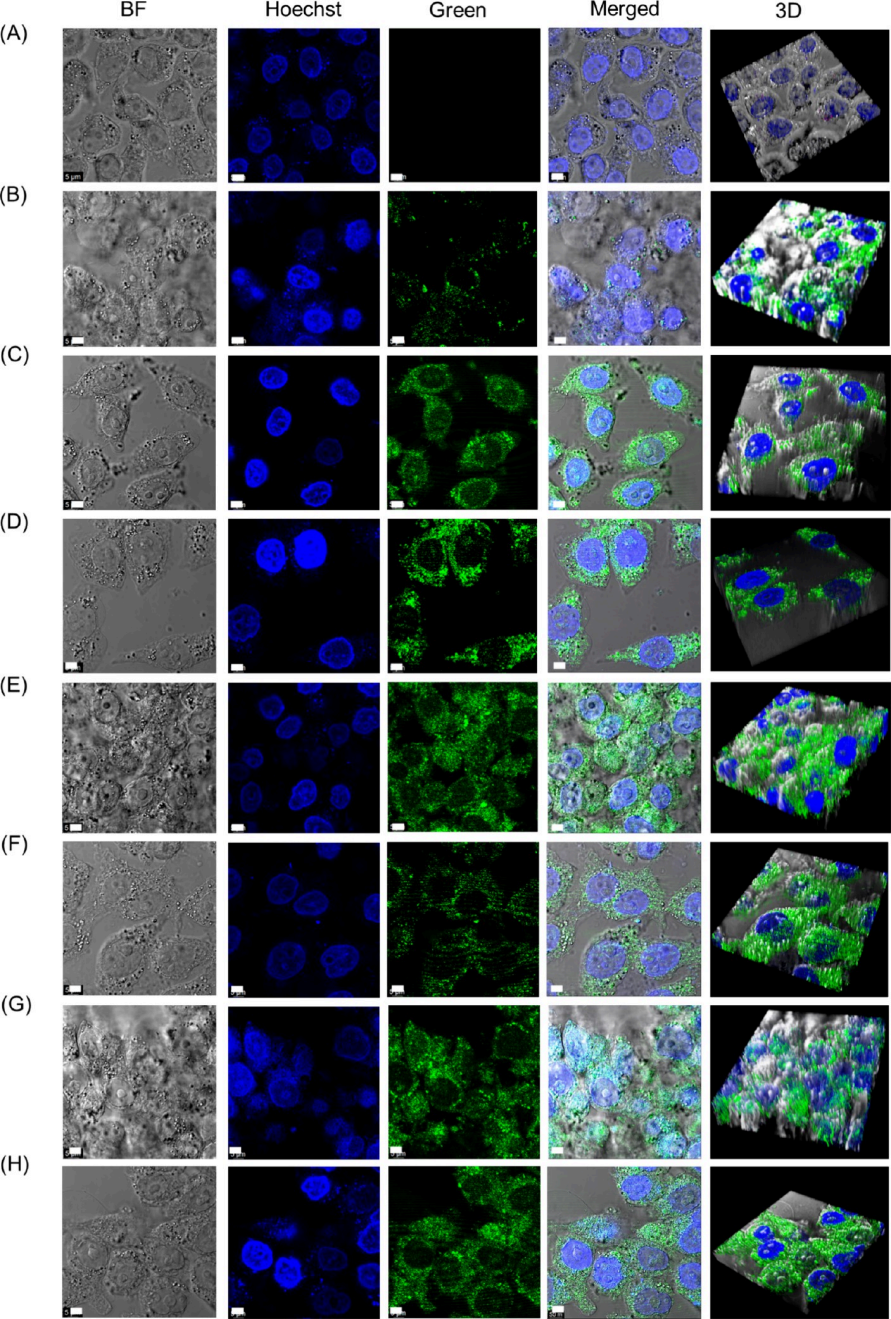
Live imaging of HeLa cells by confocal microscopy. (A) Control without any treatment. After a 24-h incubation with (B) Ac-FFWH-COO^–^, Zn^2+^, and NO_3_^–^ co-assembled with EPI, (C) Ac-FFWH-COO^–^, Zn^2+^, and NO_3_^–^ co-assembled with DOX, (D) Ac-FFWH-CONH_2_, Zn^2+^, and NO_3_^–^ co-assembled with MTX, (E) Ac-FFWH-CONH_2_, Zn^2+^, and NO_3_^–^ co-assembled with MIT, (F) Ac-FFWH-CONH_2_, Zn^2+^, and NO_3_^–^ co-assembled with 5FU, (G) Ac-FFWH-CONH_2_, Zn^2+^, and NO_3_^–^ co-assembled with CPT, (H) Ac-FFWH-CONH_2_, Zn^2+^, and NO_3_^–^ co-assembled with CP. The scale bar is 5 μm.

Following the cytotoxicity assays, we examined in vitro drug release by examining live imaging of HeLa cells, which had been incubated for 24 h with the co-assembled nanoparticles of the FFWH with each one of the drugs (Figure 8). Further pristine drugs were taken as controls to compare the uptake of co-assembled nanostructures (Figure S18). When the cells were treated with the pristine drugs, the fluorescence intensity was significantly less compared to the cells treated with the co-assembled nanostructures of the corresponding drugs, highlighting the fluorescence properties of the co-assembled nanoparticles and demonstrating the efficient uptake and release of all drugs into the cytoplasm of HeLa cells via the FFWH co-assembled carrier.

The drug localization in HeLa cells was further studied by in vitro z-stack imaging of live cells to determine the 3D projection of the cell. Overall, we can conclude that nanocarriers formed by the nanostructures of Ac-FFWH-COO^–^/-CONH_2_, Zn^2+^, and NO_3_^–^ with EPI, DOX, MTX, MIT, 5FU, CPT, and CP using the co-assembly approach, showed higher drug release in the cytoplasm, which can potentially be used for real-time optical monitoring of the drug release process.

The corresponding methodology on the drug release profiles, cytocompatibility analysis, and cellular uptake studies of FFWH drug nanoparticles is provided in M7.

## Conclusions

Overall, in this study, we used an in-house computational process to design novel minimalistic peptide nanocarriers for cancer drugs EPI, DOX, MTX, MIT, 5FU, CPT and CP. Initially, the structure of a novel minimalistic peptide scaffold of LVFH in complex with Zn^2+^ and NO_3_^–^ was elucidated and was used as a scaffold in the design. This comprised an important advantageous component in minimalistic designs. The mutations were introduced on the β-sheet domain without necessarily interfering with the β-sheet, but instead in conjunction and in compatibility with the presence of two parallel pairs of antiparallel bonded β-sheet peptides.(36) The scaffold was designed to bind a diversity of cancer drugs without the need to use or rely on drug-amino acid materialphore models, i.e., models of amino acids interacting with drugs extracted from the PDB,(61) an approach used in our previous computational studies. This comprised an additional improvement, alleviating the reliance on such modeling data and paving the way for an evolution-based computational design process with notable novel features.

Initially, multiple drug poses were initially considered, inspired by previous computational studies of our lab, capturing the lowest binding free energy for each amino acid-drug combination. The computational evolution-based design process comprised an iterative evaluation procedure, where sequential mutations in the first three residue positions were introduced based on evolution probabilities,(54) the produced designs were atomistically represented, and continuously evaluated and accepted only if there was improvement in the association free energy of drugs, as defined in our previous study.(1) We implemented a “Lock & Design as you Go” strategy with two inter-related “evolutionary” advantages considered when introducing mutations: (i) the evaluation of each design was considered on the spot, “*in processu”*, and thus, it actively affected the decision-making part process, differing from our previous studies, and (ii) a design would be locked for further improvement only if it outperformed compared to its predecessor. At the same time, histidine coordination with Zn^2+^ was considered, to allow bifunctional properties, for enhanced fluorescence. This approach also advantageously allows recurrent sequence adoption, with improvement in energy, allowing optimal structural exploration per sequence. Effectively, this could be viewed as an accelerated, modified and biased evolution-based design of peptide assemblies aiming at enhanced drug binding and maintaining Zn^2+^ coordination. Also, the evaluation metric considered within the design process comprised the average association free energy of the drugs (as defined in ref (1)) to the entire modeled scaffold (rather than an elementary structural unit), accounting “*in processu”* the effects of both deeply buried and surface exposed drugs,(1) actively guiding improved designs. The modeled scaffold was comprised of a cluster of peptides, drugs, Zn^2+^ and NO_3_^–^ (as defined in ref (1)), which in the particular study was represented by a diversity of ordered assemblies, comprising nearly perfectly ordered idealistic assemblies as well as more realistic and compact but less ordered assemblies produced by our simulations.

We identified peptides that independently achieved optimal performance across the multiple drugs considered, analogously to ref (56), and selected peptides for in-depth investigation. Four peptides, WWWH, QWWH, FFWH, and YWWH were experimentally and computationally investigated and showed high capacity to co-assemble successfully with every drug, Zn^2+^, and NO_3_^–^, forming nanocarriers. Among the four, FFWH presented high encapsulation of all the drugs according to experiments, showed a strong tendency to co-assemble with the drugs, and outperformed the rest in its ability to form β-sheet-like configurations within the simulations. Notably, its ability to form β-sheets and possess high-drug encapsulation within simulations investigating ordered assemblies is not surprising, given that it was designed to combine both properties. Interestingly, this peptide comprises the highly studied FF motif followed by an aromatic residue W and zinc-coordinating H (reviewed in ref (2)). Experimental studies showed that the FFWH peptide can co-assemble with every drug, forming nanoparticles of suitable sizes for cancer therapy. UV–vis spectroscopy assays revealed that the co-assembled nanocarriers exhibit a strong tendency to encapsulate all the drugs and demonstrated high drug release under acidic pH conditions, representing the cancer cells’ environment. Additionally, both the pristine FFWH peptides and the nanocarriers with every drug showed excellent biocompatibility with the HeLa cells. Live-cell confocal microscopy demonstrated enhanced fluorescence of the nanocarriers and efficient uptake and release of all drugs into the cytoplasm of HeLa cells via the FFWH co-assembled carrier, highlighting the FFWH co-assembled system as a promising drug delivery platform.

Our strategy comprised a computational process for designing and simulating the novel materials, balancing accuracy and efficiency, and leading to experimentally validated biological-based nanocarriers with superior properties for multiple cancer drug encapsulation compared to previous efforts. We consider that our data-free, evolution-based computational design process, coupled with simulations evaluating the designs, can serve as a paradigm for the design of novel peptide-based materials that serve as drug delivery agents in a variety of applications. In this context-the design criteria can change based on the drug delivery problem under consideration. Furthermore, additional computational studies can be used to delineate a diversity of minimalistic peptide scaffold arrangements, including peptoids and cyclic peptides. In this study, the combination of computations and experiments led to the design of a particular novel minimalistic peptide, FFWH, which can co-assemble with various cancer drugs and possess promising properties, such as enhanced fluorescence. To our knowledge, this addresses key challenges in the field.(2) Notably, the co-assembled nanocarriers, along with their corresponding structural arrangements, can be considered programmable materials, aiming to address additional challenges which are key to overcome, along with considering several additional aspects, including localization, biodistribution, biocompatibility, and efficacy of nanodrug systems *in vivo*.(2) Subsequent computational design starting from these can lead to new generations of intelligent drug delivery agents, with certain modifications and co-assembly designs,(66) aiming to provide controlled release at a specific site,(67) possess enhanced cell membrane penetration properties,(68) and exploit tumor sites’ abnormal pH profiles and pathophysiology.(67) Such new generations of advanced nanocarriers can be designed to target healthy versus cancer cells, and can be programmed to recognize particular chemokine receptors, and thus possess potential cancer and patient-specific targeting properties.

## Supplementary Material



## Supporting Information

Supporting Methods (additional details on the computational design, simulation protocols, structural and energetic analysis) and Supporting Results (supplementary figures supporting the main results) (PDF)
