# Supplementary material for: Minimalistic Peptide Nanocarriers for Multiple Cancer Drugs
Source: ACS Appl Bio Mater. 2025 Oct 6;8(10):9093–108. doi: 10.1021/acsabm.5c01234 (PMC12541707; doi:10.1021/acsabm.5c01234)
Supplement: Supplementary file 1 [file mt5c01234_si_001.pdf]

Supporting Information

Minimalistic Peptide Nanocarriers for Multiple Cancer Drugs

Anastasia Vlachou[1#], Om Shanker Tiwari[2,3,4,#], Sonika Chibh[2], Jake R. Remmert[1], Ehud Gazit[2,3,4*], Phanourios Tamamis[1,5*]

[1]Artie McFerrin Department of Chemical Engineering, Texas A&M University, College Station, Texas 77843-3122, United States.

[2]The Shmunis School of Biomedicine and Cancer Research, George S. Wise Faculty of Life Sciences, Tel Aviv University, Tel Aviv 6997801, Israel.

[3]Department of Materials Science and Engineering, Iby and Aladar Fleischman Faculty of Engineering, Tel Aviv University, Tel Aviv 6997801, Israel.

[4]Sagol School of Neuroscience, Tel Aviv University, Tel Aviv 6997801, Israel.

[5]Department of Materials Science and Engineering, Texas A&M University, College Station, Texas 77843-3003, United States.

[#] Equally Contributing First Authors

Corresponding authors:
Ehud Gazit: ehudg@post.tau.ac.il
Phanourios Tamamis: tamamis@tamu.edu

Table of Contents

- Supporting Methods ... 3

SM1. Computational Investigation of Novel Minimalistic Peptide Scaffolds in Complex with Orange G ... 3

SM1 (A). Modeling of minimalistic peptide scaffolds ... 3

SM1 (B). "Screening-like" MD simulations of minimalistic scaffolds ... 3

SM1 (C). Structural analysis of minimalistic scaffolds ... 4

SM2. Preparation of the Designable Four-Residue Peptide Scaffolds ... 5

SM2 (A). Desired Modifications on the Minimalistic Four-Residue Peptide Scaffolds for Design ... 5

SM2 (B). Preparation of the Modified Minimalistic Four-Residue Peptide Scaffolds ... 7

SM2 (C). Insertion of drugs into the modified scaffolds ... 11

SM3. Evolution-based Computational Design for all Designable Scaffolds ... 15

SM3 (A). Stages of the computational evolution-based design ... 15

SM3 (B). Consensus Peptides Identification and Ranking by Consensus Energetic Penalty 20

SM3 (C). Selected set of consensus peptides based on the "consensus energy penalty" and the aggregation propensity ... 21

SM4. Computational validation of nine selected consensus peptides - Simulating Ordered Assemblies with Different Drugs ... 23

SM4 (A). MD simulations of ordered assemblies for the selected consensus peptides ... 23

SM4 (B). Structural and Energetic analysis of the ordered assemblies ... 24

SM4 (C). Comparison of β-sheets propensity ... 25

SM5. Computational Investigation of the Top Consensus Peptides on the Early-stage Co-assembly with Different Drugs ... 26

SM5 (A). MD simulations of early-stage co-assembly for the top consensus peptides ... 26

SM5 (B). Structural Analysis of early-stage co-assembly of the top consensus peptides ... 27

SM5 (C). Structural Analysis of co-assembly for extended simulations of FFWH ... 28

SM5 (D). Association free energy of drugs and peptides with the rest of the systems on the early-stage co-assembly and in ordered assemblies ... 29

- Supporting Results ... 32
- Supporting References ... 43

Supporting Methods

SM1. Computational Investigation of Novel Minimalistic Peptide Scaffolds in Complex with Orange G

In summary, starting from the experimentally resolved structure of an amyloid-forming peptide KLVFFA from amyloid beta in complex with Orange G, we investigated truncated peptides for their capacity to represent novel minimalistic scaffolds. We studied a three-residue peptide (^{3}VFF^{5}), a four-residue peptide (^{2}LVFF^{5}), and a five-residue peptide (^{1}KLVFF^{5}) for their capacity to encapsulate Orange G and maintain the scaffold's structural integrity in comparison to ^{1}KLVFFA^{6}, which served as a control.

SM1 (A). Modeling of minimalistic peptide scaffolds.

Starting from the structure of an amyloid-forming peptide KLVFFA from Amyloid-β (Aβ) in complex with Orange G[1], UCSF Chimera was used to replicate a rectangular arrangement of (2x2x2) following the crystallographic symmetry[2]. The six-residue peptide of the initial scaffold was truncated into shorter three-residue (^{3}VFF^{5}), four-residue (^{2}LVFF^{5}), and five-residue (^{1}KLVFF^{5}) peptides in complex with Orange-G. The truncated peptide scaffolds, and additionally the control six-residue (^{1}KLVFFA^{6}) peptide scaffolds, were created through the input generator "PDB-Reader & Manipulator" of CHARMM-GUI[3,4,5,6].

SM1 (B). "Screening-like" MD simulations of minimalistic scaffolds

First, we used short simulations to investigate the capacity of the initial control six-residue peptide scaffold (^{1}KLVFFA^{6}) to encapsulate Orange G and maintain its structural integrity in accordance to the experimentally resolved structure. Subsequently, we used short "screening like" simulations to investigate the capacity of the novel minimalistic peptide scaffolds (^{1}KLVFF^{5}, ^{2}LVFF^{5} and ^{3}VFF^{5}) to encapsulate Orange G and maintain the scaffold's structural integrity in comparison to ^{1}KLVFFA^{6}, which served as a control.

The "Solution Builder" input generator of CHARMM-GUI[3,7,8] was used for the setup of the systems. All the scaffolds, composed of 48 peptides and 18 drugs, with every peptide characterized by NH_3^+ N-terminal and COO^- C-terminal, were solvated in a water cubic box of 95Å, and counter

ions of Cl^- were added, if needed, for neutrality. After the systems were prepared using all steps provided by the "Solution Builder", a short equilibration NVT simulation of 1 ns was performed in CHARMM[7], followed by short, "screening-like", 50 ns of NPT production simulations in OpenMM[9] , using the default parameters and setup provided by CHARMM-GUI[3,7,8]. The simulations performed in this section are summarized in ***Table S4 (Sim. 1.1 – Sim. 1.4).***

SM1 (C). Structural analysis of minimalistic scaffolds

Upon completion of the aforementioned simulations, Wordom[10,11] was used to calculate the percentage ratio of the radius of gyration (Å) of the scaffolds per 1 ns divided by the radius of gyration of the corresponding truncated scaffold before the simulation started[12,13]. For the calculations all the atoms were considered. According to visual inspection, all the truncated scaffolds showed nearly excellent capacity to encapsulate the Orange-G compound, similar to the control. However, among the truncated peptide scaffolds, the four-residue peptide scaffold stood out for its capacity to maintain its structural integrity with respect to the control six-residue peptide scaffold ***(Figure S1).*** The structural integrity at this stage was evaluated by the simulated systems' capacity to maintain their initial radius of gyration. This criterion was introduced using visual inspection, as particular systems (three-residue peptide and five-residue peptide) tended to expand and partially lose their integrity. Hence, the four-residue peptide scaffold represented a system that sufficed both criteria; the capacity to encapsulate Orange G and maintain its structural integrity.

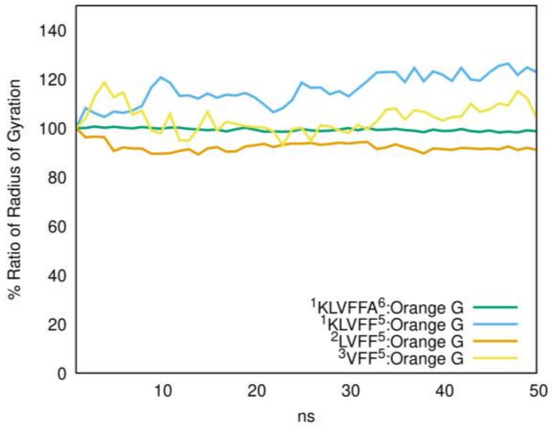

Figure S1. % Ratio of radius of gyration as a function of the simulated time for the control six-residue peptide scaffold (green), and the truncated five-residue (blue), four-residue (orange) and three-residue (yellow) peptide scaffolds.

Therefore, the four-residue peptide scaffold was selected as a basis for further design. At this point, its symmetry was increased from (2x2x2) ***(Figure S2(A))*** to a rectangular arrangement of (5x5x2) ***(Figure S2(B)),*** aiming to increase the number of drugs within the scaffold that are less accessible to the solvent. According to the new arrangement, the scaffolds hereafter are composed of 96 peptides and 40 drugs.

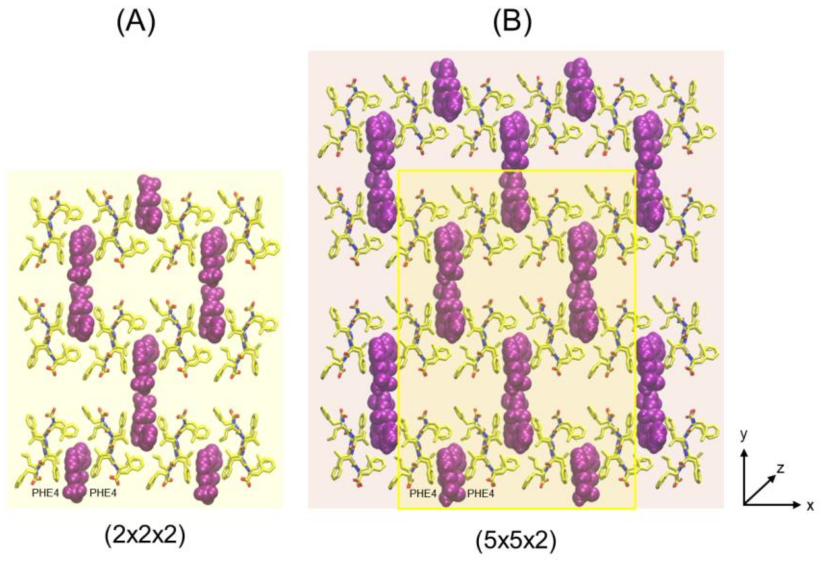

Figure S2. Molecular graphics images[14] of the ^{2}LVFF^{5}:Orange-G peptide scaffold in a rectangular arrangement of; (A) (2x2x2) and (B) (5x5x2). The peptides are shown with a yellow licorice representation and Orange-G with a purple vdW representation.

SM2. Preparation of the Designable Four-Residue Peptide Scaffolds

SM2 (A). Desired Modifications on the Minimalistic Four-Residue Peptide Scaffolds for Design.

Upon visual inspection of the ^{1}LVFF^{4}:Orange G structural unit, the fourth residue position was selected for histidine substitution, aiming at Zn^{2+} coordination. The benzyl groups of the phenylalanine residues at this position were oriented towards each other and do not participate in the binding pocket with Orange G ***(Figure S3)***. Hence, the H4F mutation was performed using the "PDB-Reader & Manipulator" input generator of CHARMM-GUI[3,4,5,6]. As shown below, the particular arrangement between the histidine residues proved to be suitable for Zn^{2+} coordination.

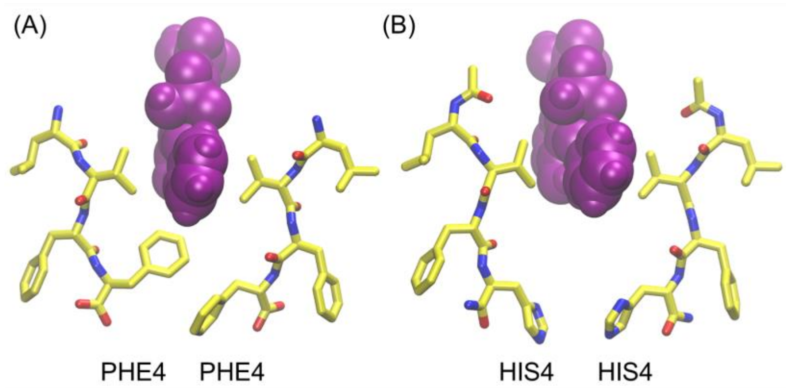

Figure S3. Molecular graphics images[14] of (A) [1]LVFF[4]:Orange G structural unit and (B) [1]LVFH[4]:Orange G structural unit. The peptides are shown with a yellow licorice representation and Orange-G with a purple vdW representation.

Due to the different net charge of the cancer drugs considered for subsequent design, different scaffolds were prepared with different compositions of peptide termini and with varying ratios and placement of Zn^{2+} and NO_3^- for charge neutrality in each system. The drugs under investigation included Epirubicin (EPI), Doxorubicin (DOX), Methotrexate (MTX), Mitomycin-C (MIT), 5-Fluorouracil (5FU), Camptothecin (CPT) and Cyclophosphamide (CP). The modifications per case were considered under the assumption that the net charge of the amino acid side chains is zero at the corresponding neutral conditions.

Therefore, we introduced Ac- and -$CONH_2$ terminals with [Peptides]:[Zn^{2+}]:[NO_3^-] = [4]:[1]:[2], a combination that yields neutral systems for scaffolds intended for neutral drugs (MIT, 5FU, CPT and CP). We employed Ac- and -COO^- terminals with [Peptides]:[Zn^{2+}] = [4]:[1], a combination that yields neutral systems for scaffolds intended for single positively charged drugs (EPI, DOX), while we employed Ac- and -$CONH_2$ terminals with [Peptides]:[Zn^{2+}] = [4]:[2], a combination that yields neutral systems for scaffolds intended for the double negative charged drug (MTX) ***(Figure S4)***. The terminals were selected after investigating Ac- and -$CONH_2$ compared to NH_3^+ - COO^- as described below.

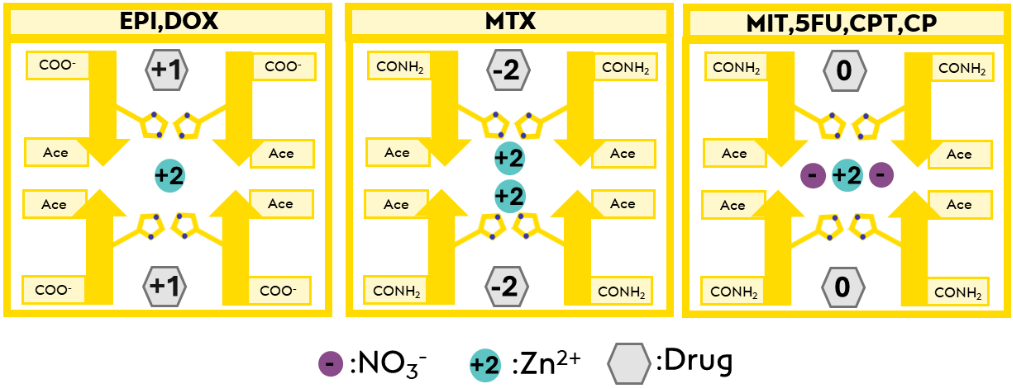

Figure S4. Illustration of a unit pocket comprising four peptides, and the different peptide termini introduced to accommodate neutrality for neutral (left), +1 positively charged (center), and -2 negatively charged (right) drug peptide nanocarriers in design.

SM2 (B). Preparation of the Modified Minimalistic Four-Residue Peptide Scaffolds

Neutral Drugs. Following the histidine mutation resulting to LVFH peptides, we introduced the Zn^{2+} in the ratio of [Peptides]:[Zn^{2+}]:[NO_3^-] = [4]:[1]:[2] using Fortran and Matlab programs as described below. Specifically, we introduced the Zn^{2+} in the center of a tetrahedron formed by the four closest histidine residues. Additionally, the NO_3^- were introduced based on the binding of NO_3^- with the histidine rings observed in the co-assembled clusters of Cyclo-HH, with Epirubicin in the presence of Zn^{2+} and NO_3; see Fig. 3B of reference[15]. Particularly, in-house Fortran programs were developed to identify all neighboring pairs of histidine residues in the scaffolds and place one Zn^{2+} in the center of a tetrahedron formed by the NE2 atoms of the four histidine residues in each neighboring pair ***(Figure S5A, S5B)***. Following that, Matlab programs were used to calculate the coordinates of the nitrogen atom of NO_3^- based on the reference distances between the NE2 atom in the histidine ring and the nitrogen atom of NO_3^-. It is worth noting that the reference distances were applied for the NE2 atoms of the two closest histidine residues, which belong to the two different neighboring pairs. The reference distances were extracted from the co-assembled clusters of Cyclo-HH, with Epirubicin in the presence of Zn^{2+} and NO_3^- (see 15 Figure 3B). Fortran programs were applied to place the nitrogen atoms of NO_3^- in the scaffolds according to the coordinates calculated from Matlab, and finally, the entire NO_3^- molecules were modeled in the pockets ***(Figure S5A, S5B).***

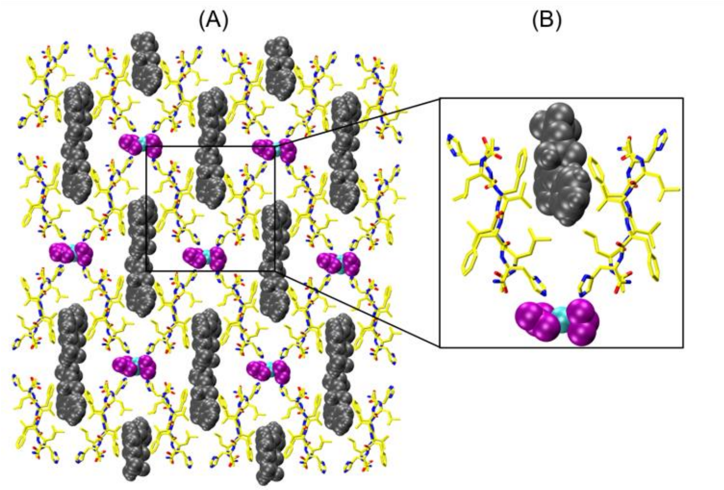

Figure S5. Molecular graphics images [14] of (A) [1]LVFH[4]:Orange G scaffold for the neutral drugs with [Peptides]:[Zn^{2+}]:[NO_3^-] ratio of [4]:[1]:[2], (B) [1]LVFH[4]:Orange G structural unit respectively. The peptides are shown with yellow licorice representation, Orange G with gray vdW, Zn^{2+} with cyan vdW and NO_3^- with purple vdW.

Upon introducing the histidine mutation as well as Zn^{2+} and NO_3^- placement in the desired ratio for neutral drugs we explored two termini combinations for the scaffold's peptides; (a) NH_3^+ - COO^-, referred to as Modified Scaffold (MS type 0), and (b) Ac - $CONH_2$, referred to Modified Scaffold (MS type 1) ***(Table S1).*** The different terminals were applied using the "PDB-Reader & Manipulator" input generator of CHARMM-GUI[3,4,5,6]. The two different combinations of termini were considered as they can affect the co-assembly properties of the system: (1) The termini are expected to affect the Zn^{2+} coordination and potentially on drug encapsulation; (2) The termini are expected to have an impact on β-sheet properties of the peptides[16,17,18,19,20].

Both scaffolds (MS type 0 and MS type 1) were further investigated via MD simulations. The set-up of the simulated scaffolds was performed using the "Multicomponent Assembler" input generator of CHARMM-GUI[3,7,8,21], according to which they were solvated in a water cubic box of 130 Å. After the systems were prepared using all steps provided by the Multicomponent Assembler, a short equilibration NVT simulation was performed, followed by an NPT production simulation in OpenMM[9], using the default parameters and setup provided by CHARMM-GUI[3,7,8,21]. The scaffolds were simulated in 3 replicates of 100 ns. Upon completion of the

simulations, the time average of the radius of gyration was calculated for every replicate in 1ns intervals using Wordom[10,11]. The average across all the replicates per simulated scaffold revealed better structural integrity for the scaffolds with the Ac - $CONH_2$ peptide terminals compared to NH_3^+ - COO^- (11.96 ± 2.05 Å compared to 16.82 ± 3.71 Å).

Thus, this led us to choose the combination of Ac - $CONH_2$ peptide terminals (MS type 1) for the scaffolds under investigation for neutral drugs. Thus, MS type 0 was discarded for any further studies, while MS 1 served as a basis for subsequent investigation involving neutral drugs. Upon completion of the simulations with the Ac - $CONH_2$ peptide scaffolds, we extracted from each replicate a single flexible template before the simulations started at 0 ns, and flexible templates at 25 ns and 100 ns time frames for further design. These were subsequently used to prepare IPS (0 ns) and SES (25 and 100 ns) scaffolds for neutral drugs.

Positively charged (+1) drugs. We utilized the same scaffolds prepared for neutral drugs (as described above) with NO_3^- ions removed, and conversion of (Ac) -$CONH_2$ terminal to COO^-. Thus, this led to modified IPS (0 ns) and SES (25 and 100 ns) scaffolds for positively charged (+1) drugs.

Negatively Charged (-2) Drugs. For negatively charged drugs, the LVFH scaffold with Ac-$CONH_2$ terminals was first simulated in the absence of ions, referred to as Modified Scaffold 2 (MS type 2) in Table S1, and then Zn^{2+} were introduced upon completion of the simulations in the extracted flexible templates at 0 ns, 25 ns and 100 ns. This was performed to avoid any excess of positive charge in the presence of a neutral compound within the designed binding pocket. Thus, this led to IPS (0 ns) and SES (25 and 100 ns) scaffolds for negatively charged (-2) drugs.

The simulations were performed in three replicates using the same conditions as described above for the other types of modified scaffolds. In the extracted templates at 25 ns and 100 ns before introducing Zn^{2+}, we performed energy minimization by constraining every pair of the closest histidine residues to enable the Zn^{2+} placement, since the goal was the ratio of [Peptides]:[Zn^{2+}] = [4]:[2]. For the constraints, we used as reference distances those extracted from the template at 0 ns. The pairs of histidine residues were identified using in-house Fortran programs, and the minimization was performed using CHARMM[7]. The energy minimization was performed by

employing 200 steps of the Steepest Descent (SD) algorithm, followed by 200 steps of the Adopted Basis Newton-Raphson (ABNR) algorithm, then repeating this sequence (200 steps of SD, 200 steps of ABNR), and concluding with 200 steps of SD. For the constraints we used a spherical harmonic potential, the Miscellaneous Mean Field Potential (MMFP) by imposing as cutoff the reference distances (~2.2 Å extracted from the template at 0 ns, which aligns with several experimentally resolved structures[22,23]) between the NE2 atoms of the histidine residues with force constant 10 kcal/mol.

The Zn^{2+} placement was performed as follows. Using Fortran codes, first, we identified the pairs of the closest histidine residues in the scaffolds. Following that, Matlab codes were used to calculate the coordinates of Zn^{2+} based on the reference distances between the NE2 atom in the histidine rings and Zn^{2+}, as well as between the CE1 atom in the histidine rings and Zn^{2+}. The reference distances were extracted from the co-assembled clusters of Cyclo-HH, with Epirubicin in the presence of Zn^{2+} and NO_3^- (see 15 Figure 3B). Inspired by our previous studies, we aimed to introduce Zn^{2+} by mimicking the geometry of the binding mode between the histidine rings of Cyclo-HH and $Zn^{2+.}$ Finally, Fortran codes were applied to impose the Zn^{2+} between each pair of closest histidine residues according to the coordinates calculated from Matlab.

The simulations performed in this section are summarized in ***Table S4 (Sim. 2.1 – Sim. 2.10).***

Table S1. Different types of minimalistic four-residue peptides, Modified Scaffolds

Four-residue peptides											
MS Type	Conditions										
	Sequence	N - terminal	C - terminal	(Peptide: OrangeG)	(Zn^{2+}: Peptide)	(NO_3^-:Zn^{2+})	# of peptides	# of Orange-G	# of Zn^{2+}	# of NO_3^-	Total Charge of Scaffold
0	LVFH	NH_3^+	COO-	1:2	(1:4)	(2:1)	96	40	14	28	0
1	LVFH	Ac	$CONH_2$	1:2	(1:4)	(2:1)	96	40	14	28	0
2	LVFH	Ac	$CONH_2$	1:2	-	-	96	40	0	0	0

SM2 (C). Insertion of drugs into the modified scaffolds

The drugs under investigation were introduced by superimposing them onto Orange-G, using the ShaEp algorithm[24] for all modified scaffolds, accordingly (i.e. neutral and positively charged were introduced to MS type 1, negatively charged drugs were introduced to MS type 2). Neutral drugs were superimposed based on their volume only, while charged drugs were based on their volume and charge. The drugs' insertion to the binding pockets was performed for all scaffolds, extracted at 0, 25 and 100 ns.

Additionally, for the templates extracted at 0 ns, which comprised nearly perfect symmetry, we developed an in-house sampling method, written in CHARMM[7], to investigate multiple poses of the imposed drugs, inspired by our lab's previous studies[25,26,27,28] that demonstrated the importance of investigating multiple orientations of a compound in a protein-binding pocket. In this study, we generated multiple poses of the drug, which ensured a representative sampling of the available scaffold units' space. For each drug, we defined two vectors (v1, v2) based on two diagonally opposite atoms ***(Table S2)***. The drug was then subjected to rotational manipulations to explore the conformational space along the unit comprehensively. Specifically, we rotated each drug in 30° intervals around the vector orthogonal to v1 and v2 for 180° (primary rotation angle, theta). For each interval, we rotated the drug around v1 in 30° sub-intervals for 360° (secondary rotation angle, phi). I.e., for each primary rotation (6 positions over 180°), we applied the secondary rotation (12 positions over 360°). By applying this two-tiered rotational approach, we systematically generated 84 unique poses for each drug. Notably, peptides and ions remained constrained in the scaffolds

during the sampling, and upon completion, we minimized the energy of the newly generated scaffolds. The energy minimization was performed using CHARMM[7] by employing 100 steps of the Steepest Descent (SD) algorithm, followed by 100 steps of the Adopted Basis Newton-Raphson (ABNR) algorithm, and concluding with 100 steps of SD.

Table S2. Selected atoms for defining the vectors v_1 and v_2 per drug.

**Drug**	**EPI**	**DOX**	**MIT**	**5FU**	**CPT**	**CP**	**MTX**
**Vector 1 (v_1)**	(O_5,O_6)	(O_5,O_4)	(N_2,N)	(N_2,N_1)	(N_2,C_{13})	(N_2,O_1)	(C_5,C_7)
**Vector 2 (v_2)**	(C_{25},C_{22})	(C_{25},C_2)	(C_{13},O_2)	(F_1,O_2)	(O_1,C_{19})	(N_1,C_2)	(N_1,N)

Upon completion of sampling and energy minimization, out of all poses per drug, we selected those which correspond to the phi-angle with the lowest van der Waals (VdW) energy per theta-angle interval, leading to seven poses per drug, i.e. seven scaffolds in total per drug. The aforementioned process resulted in seven Initially-Prepared Scaffolds (IPS_s) per drug ***(Table S3).*** Due to the lack of nearly perfect symmetry in the scaffolds extracted at 25 and 100 ns, sampling was not performed in these cases, and instead, additional energy minimization was conducted after the drugs were superimposed using CHARMM[7]. The energy minimization was performed by employing 100 steps of the Steepest Descent (SD) algorithm, followed by 100 steps of the Adopted Basis Newton-Raphson (ABNR) algorithm, and then concluding with 100 steps of SD. The aforementioned process resulted in six Simulation-Extracted Scaffolds (SES_s), from snapshots extracted at 25 ns from three triplicate runs, as well as snapshots extracted from three triplicate runs ***(Table S3)***.

In summary, IPS scaffolds provide nearly perfectly ordered assemblies but with an idealistic approach due to the truncated nature of peptides, while SES scaffolds provide slightly less ordered assemblies, which are realistic and more compact ***(Figure S6).***

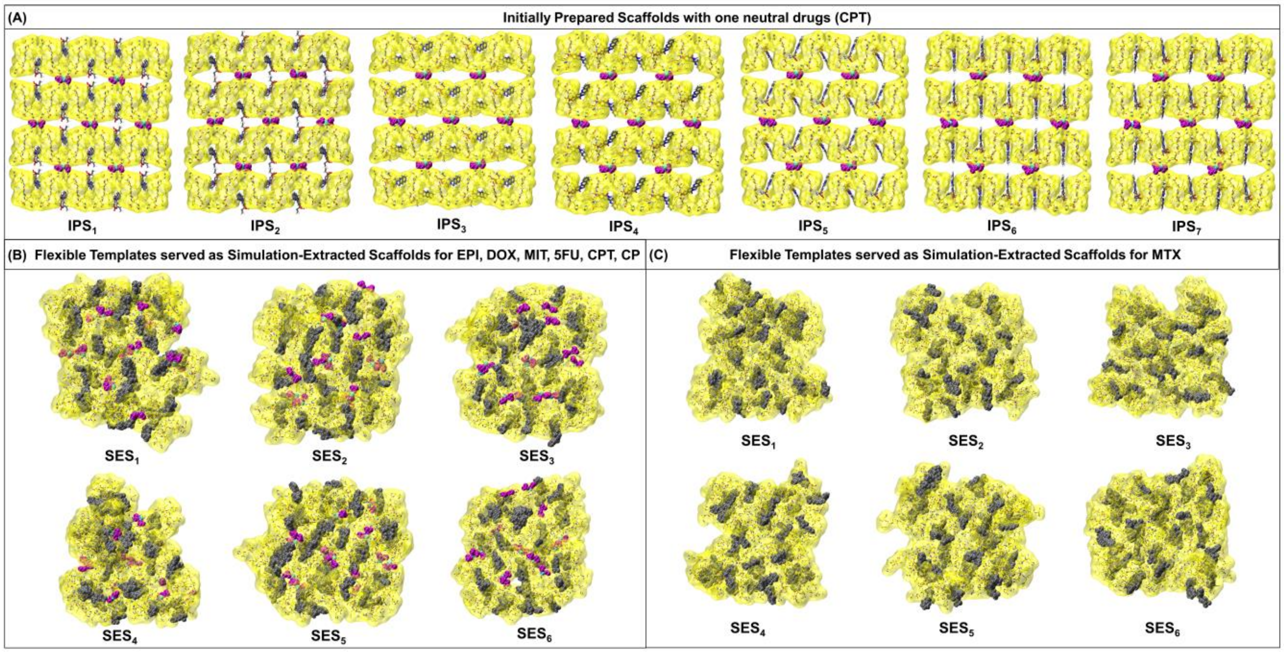

Figure S6. Molecular graphics images[14] of (A) the Initial Prepared Scaffolds with CPT (IPS_1, IPS_2, IPS_3, IPS_4, IPS_5, IPS_6, IPS_7), (B) the flexible templates extracted from simulations of MS type 1 at 25 ns (SES_1, SES_2, SES_3) and at 100 ns (SES_4, SES_5, SES_6) used neutral and positively charged drugs. (B) The flexible templates extracted from simulations of MS type 2 at 25 ns (SES_1, SES_2, SES_3) and at 100 ns (SES_1, SES_2, SES_3) were used for negatively charged drugs. The LVFH peptides are shown with yellow licorice and transparent quick surface, CPT with gray licorice, Orange-G with gray vdW, Zn^{2+} with cyan vdW and NO_3^- with purple vdW.

Table S3. Initially-Prepared Scaffolds (IPSs) and Simulation-Extracted Scaffolds (SESs)

Initially Prepared Scaffolds (IPS)														
Type of IPS	Extracted frame	Drug Pose	EPI, DOX				MIT, 5FU, CPT, CP				MTX			
			Modified scaffold	N-termini	C-termini	Zn^{2+}: Peptide	Modified scaffold	N-termini	C-termini	Zn^{2+}: Peptide	Modified scaffold	N-termini	C-termini	Zn^{2+}: Peptide
IPS_1	0 ns	$\theta = 0^o$	type:1	Ac	COO^-	(1:4)	type:1	Ac	$CONH_2$	(1:4)	type:2	Ac	$CONH_2$	(1:2)
IPS_2	0 ns	$\theta = 30^o$	type:1	Ac	COO^-	(1:4)	type:1	Ac	$CONH_2$	(1:4)	type:2	Ac	$CONH_2$	(1:2)
IPS_3	0 ns	$\theta = 60^o$	type:1	Ac	COO^-	(1:4)	type:1	Ac	$CONH_2$	(1:4)	type:2	Ac	$CONH_2$	(1:2)
IPS_4	0 ns	$\theta = 90^o$	type:1	Ac	COO^-	(1:4)	type:1	Ac	$CONH_2$	(1:4)	type:2	Ac	$CONH_2$	(1:2)
IPS_5	0 ns	$\theta = 120^o$	type:1	Ac	COO^-	(1:4)	type:1	Ac	$CONH_2$	(1:4)	type:2	Ac	$CONH_2$	(1:2)
IPS_6	0 ns	$\theta = 150^o$	type:1	Ac	COO^-	(1:4)	type:1	Ac	$CONH_2$	(1:4)	type:2	Ac	$CONH_2$	(1:2)
IPS_7	0 ns	$\theta = 180^o$	type:1	Ac	COO^-	(1:4)	type:1	Ac	$CONH_2$	(1:4)	type:2	Ac	$CONH_2$	(1:2)
Simulation-Extracted Scaffolds (SES)														
Type of SES	Extracted frame	Drug Pose	EPI, DOX				MIT, 5FU, CPT, CP				MTX			
			Modified scaffold	N-termini	C-termini	Zn^{2+}: Peptide	Modified scaffold	N-termini	C-termini	Zn^{2+}: Peptide	Modified scaffold	N-termini	C-termini	Zn^{2+}: Peptide
SES_1	25 ns	# 1	type:1	Ac	COO^-	(1:4)	type:1	Ac	$CONH_2$	(1:4)	type:2	Ac	$CONH_2$	(1:2)
SES_2	25 ns	# 2	type:1	Ac	COO^-	(1:4)	type:1	Ac	$CONH_2$	(1:4)	type:2	Ac	$CONH_2$	(1:2)
SES_3	25 ns	# 3	type:1	Ac	COO^-	(1:4)	type:1	Ac	$CONH_2$	(1:4)	type:2	Ac	$CONH_2$	(1:2)
SES_4	100 ns	# 1	type:1	Ac	COO^-	(1:4)	type:1	Ac	$CONH_2$	(1:4)	type:2	Ac	$CONH_2$	(1:2)
SES_5	100 ns	# 2	type:1	Ac	COO^-	(1:4)	type:1	Ac	$CONH_2$	(1:4)	type:2	Ac	$CONH_2$	(1:2)
SES_6	100 ns	# 3	type:1	Ac	COO^-	(1:4)	type:1	Ac	$CONH_2$	(1:4)	type:2	Ac	$CONH_2$	(1:2)

SM3. Evolution-based Computational Design for all Designable Scaffolds

SM3 (A). Stages of the computational evolution-based design

The evolution-based computational design process was applied for all drugs under investigation, independently. For every drug, all the corresponding Initially Prepared Scaffolds (IPS_1 to IPS_7) and all the Simulated Extracted Scaffolds (SES_1 to SES_6) were given as inputs to the evolution-based design process ***(Figure S7(A))***. Each iteration consists of two stages, described below:

Stage 1 – Introduction of Mutations at positions 1, 2 and 3 *(Figure S7(B))*. For each mutable position (1, 2, and 3), a random amino acid was selected from the 20 standard amino acids. Each mutation per position was deemed acceptable based on the probability of the previous locked amino acid to be mutated to the new one in comparison with the respective probability of the PAM250 evolution matrix.[29] This could be considered as an accelerated mutation process, inspired by the evolution probabilities provided by the PAM250 matrix. This approach can lead to possible combinations of new sequences that can have no new amino acids, one new amino acid at any position, two new amino acids at any position or all new amino acids. However, only sequences with at least one new amino acid were accepted; otherwise, the introduction of mutations was repeated until a sequence with at least one new amino acid was produced. Furthermore, sequences with any of CYS, PRO or GLY were rejected to avoid any potential disulfide bridge formation, and any sequences not promoting β-sheet formation.[30,31,32] Lastly, a condition was introduced to ensure neutrality of the produced sequence. Due to the three amenable positions for modifications, an introduction of a positively (ARG or LYS) / negatively (GLU or ASP) charged residue at one position should accommodate a concurrent introduction of a negatively/positively charged residue at any of the other two positions; otherwise, a designs was not considered acceptable and the introduction of new mutations was repeated. Then, the scaffold structure was regenerated through energetic minimization with backbone adaptation and sidechain replacement according to the new acceptable sequence. Particularly, we employed 200 steps of the Steepest Descent (SD) algorithm, followed by 200 steps of the Adopted Basis Newton-Raphson (ABNR) algorithm, then repeating this sequence (200 steps of SD, 200 steps of ABNR), and concluding with 200 steps of SD using CHARMM[7] programs originated from CHARMM-GUI[3,4,5,6], and were modified accordingly. By the end of Stage 1 of each iteration, a new acceptable design has been produced. Stage 1 consisted of in-house FORTRAN and Unix scripts.

Stage 2 – Energy Evaluation *(Figure S7(C))*. For any new acceptable design produced in each iteration, the association free energy of each drug molecule with the rest of the scaffold system[15] was calculated using the empirical scoring function Vinardo[33], and then the average was calculated across all drug molecules and used as the evaluation metric. The files were prepared using OpenBabel[34].

The two stages in each iteration were followed by the **"Lock & Design as you Go" criterion *(Figure S7(D))*.** According to this, if the energy value of the new acceptable design was lower than the minimum of the energy values of all the produced acceptable designs so far, then the current design was "locked" as the best so far and given as input to stage 1 of the next iteration. The "***Lock & Design as you Go***" criterion was applied using in-house LINUX scripts developed in this study. For each designable scaffold per drug, the evolution-based design was performed in three independent runs. Thus, in total, $(7(IPS_{1...7}) + 6\ (SES_{1...6}))*3 = 39$ runs were performed for each drug. The iterations proceeded for a minimum of 600 cycles, with additional extensions of 100 iterations for systems that did not exhibit convergence, defined as the absence of a new lock within the last 100 iterations.

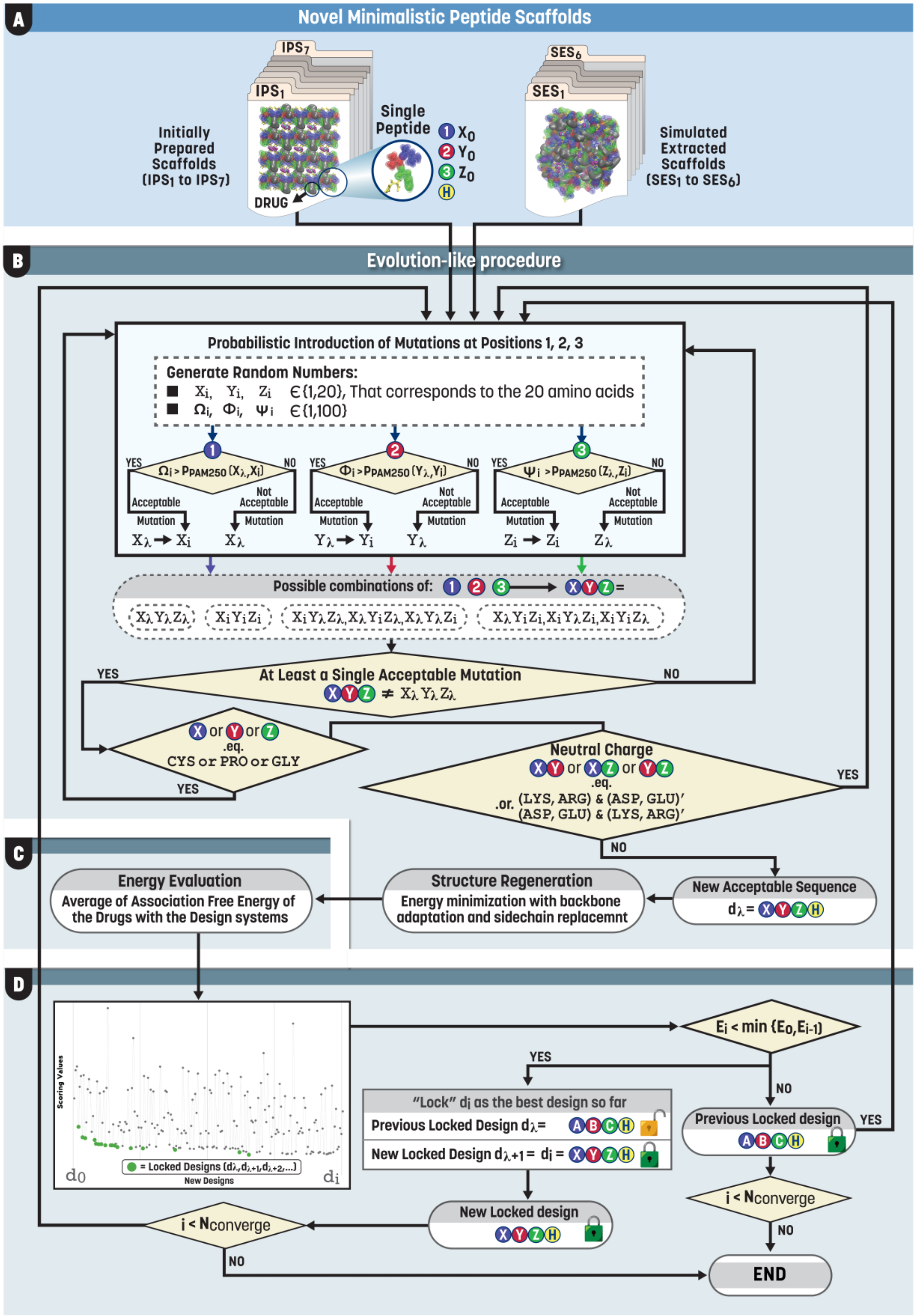

A
Novel Minimalistic Peptide Scaffolds
Initially Prepared Scaffolds (IPS1 to IPS7)
Single Peptide
DRUG
Simulated Extracted Scaffolds (SES1 to SES6)
B
Evolution-like procedure
Probabilistic Introduction of Mutations at Positions 1, 2, 3
Generate Random Numbers:
Xi, Yi, Zi ∈{1,20}, That corresponds to the 20 amino acids
Ωi, Φi, Ψi ∈{1,100}
Acceptable Mutation
Not Acceptable Mutation
Possible combinations of:
At Least a Single Acceptable Mutation
CYS or PRO or GLY
Neutral Charge
(LYS, ARG) & (ASP, GLU)'
(ASP, GLU) & (LYS, ARG)'
C
Energy Evaluation
Average of Association Free Energy of the Drugs with the Design systems
Structure Regeneration
Energy minimization with backbone adaptation and sidechain replacemnt
New Acceptable Sequence
D
Scoring Values
Locked Designs
New Designs
"Lock" di as the best design so far
Previous Locked Design
New Locked Design
Previous Locked design
New Locked design
END

Figure S7. Overview of the evolution-based design process used for the design of tetra-peptides with improved drug's association energy as it was applied for every drug under investigation. (A) **Novel Minimalistic Four-Residue Peptide Scaffolds:** were classified into two categories based on the way they were derived (see Methods 1A); Initially Prepared Scaffolds (IPS_1 to IPS_7) and Simulation-Extracted Scaffolds (SES_1 to SES6), and all of them were subjected (used as input) for design. Two molecular graphics images of one representative *Initially Prepared Scaffold* and one *Simulation-Extracted Scaffold* were used to indicate the different residue positions. The residues are shown in licorice and transparent vdW representation in different colors; blue, red, green and yellow, correspond to residues 1, 2, 3 and 4, respectively. A histidine residue at position 4 is not subject to any changes during design, while residue positions 1, 2 and 3 are symbolized with X_o, Y_o and Z_o, and initially correspond to L, V and F amino acids, respectively. An example drug is shown in gray quick surface representation[14]. (B) **Evolution-Based Design Process:** for every designable scaffold, the process was repeated for i-iterations until convergence ($i < N_{convergence}$). Every iteration started with the *Probabilistic Introduction of Mutations* at positions 1, 2 and 3. For each mutable position, a new amino acid was randomly selected (represented with the X_i, Y_i and Z_i Є {1,20} respectively). Each mutation per position was deemed acceptable only if a randomly generated number Ω_i, Φ_i and Ψ_i Є {1,100} was higher than the probability of the previous locked amino acid at the specific position (X_λ, Y_λ and Z_λ) to be mutated to the current generated (X_i, Y_i and Z_i) in comparison to probabilities from the PAM250 evolution matrix.[29] This approach of mutations can lead to possible combinations of new sequences that can have no new amino acids $\{X_iY_iZ_i\}$, one new amino acid at any position $\{X_iY_\lambda Z_\lambda, X_\lambda Y_i Z_\lambda, X_\lambda Y_\lambda Z_i\}$, two new amino acids at any positions $\{X_iY_iZ_\lambda, X_iY_\lambda Z_i, X_\lambda Y_iZ_\lambda\}$ or all new amino acids $\{X_iY_iZ_i\}$. However, only sequences with *at least a single new amino acid* were accepted; otherwise, the introduction of mutations was repeated until a sequence with at least one new amino acid was produced. Furthermore, sequences with any of CYS, PRO or GLY were rejected to avoid any potential disulfide bridge formation, and any sequences not promoting β-sheet formation[30,31,32]. Lastly, a condition was introduced to ensure *neutral charge* of the produced sequence. Due to the three amenable positions for modifications, an introduction of a positively (ARG or LYS) / negatively (GLU or ASP) charged residue at one position should accommodate a concurrent introduction of a negatively/positively charged residue at any of the other two positions; otherwise, the designs was not considered acceptable and the introduction of new mutations was repeated untill this condition was satisfied.

By the end of the mutations stage of each iteration "i", a *New Acceptable Sequence* has been produced (d_i=XYZH), according to which the *Structural Regeneration* of the scaffold was performed, including energy minimization with backbone adaptation and sidechain regeneration, leading to a new design. (C) The new design followed **"Energy Evaluation"** which was calculated as the average association free energy of all drugs with the rest of the scaffold system. (D) The new design after it was generated and evaluated (see a representative graph at which the green points represented the locked designs) were examined based on the **"Lock and Design as you Go"** criterion, according to which only if the scoring value of the current design (E_i) was less than the minimum of all the previous acceptable designs ($E_i < \min\{E_0, E_{(i-1)}\}$) then the current design (d_i=XYZH) was *locked* in the position of the previous locked design (d_λ=ABCH) as the best design so far and was given as input for introducing new mutations during the next iteration i+1. If $E_i > \min\{E_0, E_{(i-1)}\}$, then the previous locked design (d_λ=ABCH) was given as input for the next iteration i+1. The iterations proceeded for a minimum of 600 cycles, with additional extensions of 100 iterations for systems that did not exhibit convergence, defined as the absence of a new lock within the last 100 iterations ($N_{converge}$). The evolution-based design process was performed for all the designable scaffold systems of every drug under investigation in three independent runs per scaffold system. The N- and C- termini along with the ion ratio within the scaffold systems, were described in detail above.

SM3 (B). Consensus Peptides Identification and Ranking by Consensus Energetic Penalty

Per drug under investigation, the evolution-based design process was performed on every designable scaffold system

(i = "IPS1","IPS2","IPS3","IPS4","IPS5","IPS6","IPS7""SES1","SES2","SES3","SES4","SES5","SES6").

In each iteration $(n = 1 \ldots \text{Nconverge})$ we recorded each acceptable and not necessarily locked design (d_n) and its corresponding Average Association Free Energy between the drugs and the rest scaffold systems (E_n).

$$R = \{(d_1^i, E_1^i), (d_2^i, E_2^i), \ldots, (d_n^i, E_n^i)\} \mid 1 \leq n \leq \text{Nconverge}$$

$$\forall\, i \in \{IPS_1, IPS_2, IPS_3, IPS_4, IPS_5, IPS_6, IPS_7, SES_1, SES_2, SES_3, SES_4, SES_6\}$$

$$(d, E): (\text{design, Association Free Energy})$$

Importantly, the same design could have been produced in multiple instances across a design run, and across the different 39 design runs per drug. At this stage, we identified the unique entry per design per drug among all of its appearances with the lowest association free energy.

$$D_{unique} = \{d_u^i \mid u = \text{unique \# of designs}\}$$

$$E_{min} = \min\{E_u^i \mid d_u^i \in D_{unique}\}$$

$$R_{unique} = \{(d_1^i, E_{min_1}^{\;i}), (d_2^i, E_{min_2}^{\;i}), \ldots, (d_m^i, E_{min_m}^{\;i}) \mid 1 \leq m \leq u\}$$

Next, we aimed to identify the consensus best designs across all drugs under investigation, by defining a relative Association Free Energy (ΔE) with respect to the design with the lowest association free energy for each drug $(d_{top}^i, \Delta E_{top}^i = 0)$. Thus, our data were re-arranged in terms of ΔE as follows:

$$E_{top}^i = \{E_{min_1}^{\;i}, E_{min_2}^{\;i}, \ldots, E_{min_m}^{\;i} \mid d_u^i \in D_{unique} \;\&\; i \in \{AS_1, AS_2, AS_3, AS_4, AS_6, AS_7\}\},$$

$$\Delta E_m^i = E_{min_m}^{\;i} - E_{top}^i$$

This resulted in a comprehensive dataset containing the designs and the relative association free energy $(\Delta R_{unique}^{DRUG1}, \Delta R_{unique}^{DRUG2}, \Delta R_{unique}^{DRUG3}, \Delta R_{unique}^{DRUG4}, \Delta R_{unique}^{DRUG5}, \Delta R_{unique}^{DRUG6}, \Delta R_{unique}^{DRUG7})$,

where ΔR_{unique} per drug can be expressed as:

$$\Delta R_{unique} = \{(d_1^i, \Delta E_1^i), (d_2^i, \Delta E_2^i), \ldots, (d_m^i, \Delta E_m^i) \mid 1 \leq m \leq u\}$$

Finally, the common peptides across all drugs were ranked based on a "consensus energetic penalty" term, defined as the total sum of their relative Association Free Energy values (ΔE) over all drugs.

$$D^{g}_{unique} = \{d^{i}_{u} \mid u = \text{unique \# of designs}\}\ \forall\ g \in \{DRUG_1, DRUG_2, DRUG_3, DRUG_4, DRUG_5, DRUG_6, DRUG_7\}$$

$$D_{consensus} = D^{DRUG_1}_{unique} \cap D^{DRUG_2}_{unique} \cap D^{DRUG_3}_{unique} \cap D^{DRUG_4}_{unique} \cap D^{DRUG_5}_{unique} \cap D^{DRUG_6}_{unique} \cap D^{DRUG_7}_{unique}$$
$$= \{d_1, d_2, \ldots, d_c \mid c = \text{\# of consensus designs}\}$$

$$\text{Consensus Energetic Penalty} = \text{sum}(\Delta E_d) = \sum_{g=1}^{7} \Delta E_{d,g}\ \forall\ d \in D_{consensus}$$

The consensus designed peptides ranked by the *consensus energy penalty* are presented in **Figure 1.**

SM3 (C). Selected set of consensus peptides based on the "consensus energy penalty" and the aggregation propensity

At this stage we aimed at making a short list of selected consensus peptides. The list of top 20 designs contains peptides of the following pattern: hydrophobic/polar/aromatic in the first position, followed by an aromatic residue at position 2, and predominantly tryptophan (with one exception) at position 3. Peptides with the lowest consensus energetic penalty ranking comprise YWWH, FWWH, HWWH, WWWH, YYWH, LWWH, IWWH, and QWWH and were investigated in what follows. Also, from the list of top 20, the following remaining peptides, VWWH, WFWH, and FFWH were also investigated in what follows due to their highest aggregation propensity according to PASTA 2.0[35] compared to the remaining ones (WYWH, YFWH, FYWH, TWWH, MWWH, NWWH, YWYH, SWWH, AWWH ***(Figure S8 and S9(A))***.

The three peptides VWWH, WFWH, and FFWH were subjected to relatively short MD simulations for their capacity to form β-sheet-like configurations within aggregated clusters, which indicated that FFWH is more prone to aggregate and form β-sheet-like configurations compared to the rest (Methods and Results are presented below). This may not be surprising given the high-propensity of FF to self-assemble into β-sheet-like configurations.

To investigate the ability of the three peptides (VWWH, WFWH, FFWH), to self-assemble in β-sheet-like configurations we performed relatively short MD simulations of multiple copies of the same peptide, starting from a random initial arrangement. The initial structures of the peptides were generated using PEP-FOLD 3.5[36] and the first model was extracted. To meet the requirements of the PEP-FOLD software, which can build peptides of at least five amino acids, an additional glycine (GLY) was added at the N-terminus of each peptide and subsequently removed after the model generation and prior to preparing the simulation. For each peptide under investigation, multiple copies of peptides were allowed to co-assemble in the presence of ions and a solvent, as described below. The "PDB-Reader & Manipulator" input generator of CHARMM-GUI[3,4,5,6] was first used to adjust the peptide terminals (Ac- and -$CONH_2$). Following the "Multicomponent assembler" input generator of CHARMM-GUI[3,7,8,21] was used for the set-up of the simulated systems, where 48 peptide copies, 12 Zn^{2+} ions, and 24 NO_3^- ions were solvated in an 83 Å cubic box with 95:5 IPA/DMF. After the systems were prepared using all steps provided by the multicomponent assembler, a short equilibration NVT simulation was performed, followed by a long NPT production simulation in OpenMM[9], using the default parameters and setup provided by CHARMM-GUI[3,7,8,21]. Each system was simulated for 1 μs. The simulations performed in this section are summarized in ***Table S4 (Sim. 3.1 – Sim 3.3).***

Upon completion of simulations, we used in-house Fortran programs to identify the clusters comprising different components of peptides and ions, similar to our previous study[15]. A 3.5 Å distance cutoff was set to identify interacting atom pairs among different peptides and ions. If the distance between any atom pair from different peptides or ions was below this cutoff value, those particular peptides or ions were considered part of the same cluster. Notably, the periodic boundary conditions were considered in the identification of interacting atom pairs and therefore in the clusters detection and representation as well. Upon detection of clusters, their size was defined as the sum of all interacting entities, peptides and ions, respectively. The analysis was performed for every 1 ns increment, at which we calculated: (a) the number of the formed clusters ***(Figure S9(B))*** and (b) the percentage probability of peptides to form β-sheet-like configurations within aggregated clusters as the fraction of the number of peptides in β-sheet-like configurations divided by the total number of the peptides in the cluster ***(Figure S9(C))***. The definition of the β-sheet-like configurations, which were incorporated in the Fortran programs, was based on this approach.[37] The average value across all the clusters of the same clusters' size bin, along with population

standard deviations, was plotted as a function of clusters' size ***(Figure S9(C))***. The clusters were grouped into bins with a size range of 10 for analysis.

The three peptides (VWWH, WFWH, FFWH) shared a similar propensity to form aggregates (Figure S9(B)), while FFWH is the one most prone to form β-sheet-like configurations within aggregated clusters (Figure S9(C)). The large standard deviations are a consequence of the fact that some aggregated clusters, most presumably due to the relatively short time duration of the particular runs, may not necessarily contain β-sheet-like configurations, while other clusters comprise β-sheet-like configurations. Notably, we aimed to select one rather than multiple peptides from the above list, and thus FFWH, which performed to some extent better than VWWH, was selected only for the sake of reducing the list of peptides for further investigation. In what follows, the investigation focuses on validating the following nine peptides: YWWH, FWWH, HWWH, WWWH, YYWH, LWWH, IWWH, QWWH, and FFWH, which were referred to as selected consensus peptides.

SM4. Computational validation of nine selected consensus peptides - Simulating Ordered Assemblies with Different Drugs

We first used MD simulations to study whether *selected consensus peptides*, co-assembled with drugs and ions, can maintain the integrity of the ordered assemblies they were designed based upon, originating from both IPS and SES scaffolds (***Table S4; Sim. 4.1 – Sim. 4.126***). Upon completion of simulations, structural and energetic analysis were performed. Additionally, based on a variation observed in the consensus association free energy of some peptides with the rest, the early-stage self-assembly properties of these peptides were investigated further (***Table S4; Sim.127 – Sim 4.131***).

SM4 (A). MD simulations of ordered assemblies for the selected consensus peptides

We first identified for each drug, the IPS and SES corresponding systems with the lowest association free energy. As mentioned above, IPS scaffolds provide nearly perfectly ordered assemblies but with an idealistic approach due to the truncated nature of peptides, while SES scaffolds provide slightly less ordered assemblies, which are realistic and more compact. Thus, we considered it important to study designs stemming from the lowest association free energy system of both approaches. Per drug, both systems were simulated. The systems were prepared using the

"Multicomponent Assembler" input generator of CHARMM-GUI[3,7,8,21]. The systems were solvated in solvent conditions of (IPA:DMF)=(95:5), and counter ions of Na+ were added if needed for neutrality. The size of the box was defined for each case by using the "Solution Builder" input generator from CHARMM-GUI[3,7,8], where we introduced 15Å for edge distance. After the systems were prepared using all steps provided by the Multicomponent Assembler, a short equilibration NVT simulation was performed, followed by a long NPT production simulation in OpenMM9, using the default parameters and setup provided by CHARMM-GUI[3,7,8,21]. Each scaffold was simulated for 100 ns. The simulations of ordered assemblies for all combinations between every peptide from the nine selected consensus peptides and every drug, originating from both IPS and SES are summarized in ***Table S4 (Sim. 4.1 – Sim. 4.126).***

SM4 (B). Structural and Energetic analysis of the ordered assemblies

Upon completion of the simulations described above, we used in-house Fortran programs to calculate the percentage of drug encapsulation within the scaffold for every 1 ns increment. To identify the peptides, drugs, and ions that are part of the scaffold in each analyzed snapshot, we used in-house Fortran programs which followed the same approach as in our previous work to identify formed multicomponent clusters[15]. Within the simulations, we observed that the systems were highly stable. The overall stability served as a first point of validation, showing that the designed peptides can adopt the structures they were designed based upon, and thus, emphasis was placed on drug encapsulation. Any minor exceptions of external peptides or drugs at the outer surface, which were seldom less stable, were identified from the Fortran programs and excluded from subsequent calculations. Hereafter these peptides per case were excluded from any calculations performed for the ordered assemblies systems.

The percentage of drug encapsulation was calculated as the fraction of the number of drugs encapsulated in the scaffold divided by the total number of drugs available in the simulated system. For every simulated scaffold, the average drug encapsulation over the simulated time was calculated and plotted. ***(Figure S10)***.

Upon verifying all selected consensus peptides' stability, we observed a nearly perfect drug encapsulation of the charged drugs, EPI, DOX and MTX with variations across the neutral drugs ***(Figure S10)*** which revealed the importance of considering more criteria to choose the

outperforming ones for further computational and experimental investigation, such as the energetic stability and the aggregation propensity.

To investigate the energetics of drug encapsulation within the simulation, we focused on the eight innermost binding pockets ***(Figure S11(A))***, and per drug-peptide combination, we considered the run (either originating from IPS or SES) with the lowest average association free energy to the system. This was performed for the final simulation snapshot per trajectory. To compare against different peptides, we identified the peptide with the lowest sum of average association free energy for all drugs (WWWH, defining at as base 0 kcal/mol) and then plotted the relative sum of average association free energies of different peptides with respect to the one corresponding to WWWH ***(Figure S11(B))***. At this stage, Autodock4Zn[38] was used for energy calculations. The calculation shows a variation between WWWH, QWWH, FFWH, HWWH and YWWH and the rest.

SM4 (C). Comparison of β-sheets propensity

The five peptides WWWH, QWWH, FFWH, HWWH and YWWH were investigated for their propensity to form β-sheet-like configurations. The peptides were built using PEP-FOLD 3.5[36], analogously to the methods outlined in SM3(C). We performed MD simulations of multiple copies of the same peptide, starting from a random initial arrangement, similarly to section SM3(C). These simulations are summarized in ***Table S4 (Sim. 4.127 – Sim. 4.131).*** Notably, the simulation comprising the FFWH peptide is the same as those performed in section SM3(C), ***Table S4 (Sim. 3.3).***

Upon completion of 1 μs simulations per system, and upon detecting the peptides in β-sheet-like configurations within the aggregated clusters (analogously to SM3(C)), we calculated the time average probability of a peptide to form β-sheet-like configurations within clusters. The probability corresponds to the sum of peptides in β-sheet-like configurations within aggregated clusters divided by the total number of peptides in the cluster, with HWWH underperforming ***(Figure S11(C))***.

Peptides WWWH, QWWH, FFWH, and YWWH are further investigated computationally and experimentally, and are referred to as *top consensus peptides*.

SM5. Computational Investigation of the Top Consensus Peptides on the Early-stage Co-assembly with Different Drugs

SM5 (A). MD simulations of early-stage co-assembly for the top consensus peptides

At this stage, we investigated the early-stage co-assembly properties of the top consensus peptides, with Zn^{2+} and NO_3^- in the presence of drugs, in comparison to the absence of drugs. The top consensus peptides were built using PEP-FOLD 3.5[36], analogously to the methods outlined in SM3(C). Two variations of the peptides were constructed with different terminal groups using the "PDB-Reader & Manipulator" input generator of CHARMM-GUI[3,4,5,6] : (1) Ac-, COO- (intended for positively charged drugs) and (2) Ac-, $CONH_2$ (intended for neutral or negatively charged drugs).

The simulations of the systems in presence of drugs were performed for 2 μs (***Table S4; Sim. 5.1 - Sim. 5.28***). The simulations of the systems including -$CONH_2$ in the absence of drugs (***Table S4; Sim. 5.29 – Sim. 5.32***) constitute an extension of those performed in SM4(C) ***(Table S4; Sim. 4.127 – Sim. 4.129 & Sim 4.131)***. The simulations of the systems including -COO^- in the absence of drugs were performed for 2 μs (***Table S4; Sim. 5.35 – Sim. 5.36***).

To investigate the ability of the top consensus peptides to co-assemble with drugs, Zn^{2+} and NO_3^- , we simulated multiple copies of each peptide, with multiple copies of the drugs, in the presence of ions. The copies of the peptides, drugs, and ions were initially placed in a random arrangement, maintaining the specific ratio determined during the design model for each case of drug. The "Multicomponent assembler" input generator of CHARMM-GUI[3,7,8,21] was used for the setup of the systems. For the positively charged drugs (EPI,DOX), the systems were composed by 48 copies of the Ac-, COO- peptides, 24 copies of the drugs and 12 Zn^{2+} ions (with respect to to the designed ratio peptides:drugs:Zn^{2+}=4:2:1). For the negatively charged drug (MTX) the system was composed by 48 copies of the Ac-, $CONH_2$ peptides, 24 copies of the drugs and 24 Zn^{2+} ions (with respect to to the designed ratio peptides:drugs:Zn^{2+}=4:2:2). For the neutral drugs, the systems were composed by 48 copies of the Ac-, CONH2 peptides, 24 copies of the drugs, 12 Zn^{2+} ions and 24 NO_3^- ions (with respect to the designed ratio peptides:drugs:Zn^{2+}:NO_3^-=4:2:1:2). All the systems were solvated in an 83 Å cubic box with 95:5 IPA/DMF. After the systems were prepared using all steps provided by the multicomponent assembler, a short equilibration NVT simulation was performed, followed by a longer NPT production simulation in OpenMM[9], using the default

parameters and setup provided by CHARMM-GUI[3,7,8,21]. Each system was simulated for 2 μs. These simulations are summarized in ***Table S4 (Sim. 5.1 – Sim. 5.28).***

Similar simulations were performed to investigate the ability of the top consensus peptides to self-assemble in the absence of drugs, analogously to SM3(C). The "Multicomponent assembler" input generator of CHARMM-GUI[3,7,8,21] was used for the setup of the systems. In the Ac-, $CONH_2$ peptide systems, 48 peptide copies, 12 Zn^{2+} ions, and 24 NO^{3-} ions were solvated in an 83 Å cubic box with 95:5 IPA/DMF. Similarly, the Ac-, COO- peptide systems contained 48 peptides and 24 Zn^{2+} ions in the same solvent and box conditions. After the systems were prepared using all steps provided by the multicomponent assembler, a short equilibration NVT simulation was performed, followed by a longer NPT production simulation in OpenMM[9], using the default parameters and setup provided by CHARMM-GUI[3,7,8,21]. Each system was simulated for 2 μs. These simulations are summarized in ***Table S4 (Sim. 5.29 – Sim. 5.36).***

SM5 (B). Structural Analysis of early-stage co-assembly of the top consensus peptides

Upon completion of simulations, we used in-house Fortran programs to identify the clusters comprising different components of peptides, drugs and ions (analogously to SM3(C)). The analysis was performed for every 1 ns increment. Upon clusters' detection, their size was defined as the sum of all interacting entities, peptides, drugs and ions, respectively, and the structural measures described below were calculated as a function of the clusters' size. The data in the graphs represent the average values with population standard deviations, plotted as a function of cluster size. The clusters were grouped into bins with a size range of 10 for analysis ***(Figure S12)***.[15]

In what follows, we provide an outline of calculations that were performed only for clusters with at least one drug encapsulated[15]:

- The **percentage of drug encapsulation** for each cluster was calculated as the fraction of the number of drugs encapsulated in the cluster divided by the total number of drugs available in the simulated system ***(Figure S13)***.
- The **percentage composition per component** (peptide, drug, Zn^{2+}, and NO_3^-) for each cluster was calculated as the fraction of the number of peptides, drugs and ions in the cluster, respectively, divided by the sum of the total number of peptides, drugs and ions comprising the cluster ***(Figure S14)***.

It's worth noting that the largest possible clusters size formed in the simulated systems including EPI or DOX couldn't be more than 84 molecules (48 peptides, 24 drug molecules and 12 Zn^{2+}), for MTX up to 96 molecules (48 peptides, 24 drug molecules and 24 Zn^{2+}) and for all the rest up to 108 molecules (48 peptides, 24 drug molecules, 12 Zn^{2+} and 24 NO_3^-).

Additionally, we calculated the **time average probability of a peptide to form β-sheet-like configurations within clusters** (analogously to SM4(C)). These calculations were performed for each of the four peptides in the presence of drugs (seven systems per peptide) and absence of drugs. Additionally, we present an average value of each peptide across the seven different drugs ***(Figure S15).***

SM5 (C). Structural Analysis of co-assembly for extended simulations of FFWH

FFWH outperformed the rest in its enhanced propensity to co-assemble into β-sheet-like configurations in the presence of drugs and ions, with the exception of EPI (***Figure S15***), and thus, we extended the simulations of the particular peptide in the presence of drugs up to 4 μs in total per system (***Table S4, Sim. 5.35 – Sim. 5.45*** are extensions ***of Sim. 5.15 – Sim. 5.21***). At this stage, we provide an outline of additional calculations that were performed only for clusters with at least one drug encapsulated ***(Figure 1)***[15]:

- The **percentage of Zn^{2+} encapsulation** for each cluster was calculated as the fraction of the number of Zn^{2+} encapsulated in the cluster divided by the total number of Zn^{2+} available in the simulated system.
- The **percentage of peptide encapsulation** for each cluster was calculated as the fraction of the number of peptides encapsulated in the cluster divided by the total number of Zn^{2+} available in the simulated system.
- The **probability of a drug molecule to mediate interactions between two peptides** for each given cluster, referred as DPP (where D: is a drug, P: is a peptide) was calculated as the fraction of the number of interactions at which the middle is a drug (D) mediating two different peptides (P), divided by the total number of drugs in the cluster.
- The **probability of a drug molecule to mediate interactions between two peptides and Zn^{2+}** for each given cluster, referred as DPZP (where D: is a drug, P: is a peptide, Z: is a zinc)

was calculated as the fraction of the number of interactions at which the middle is a drug (D) mediating two peptides (P) and one zinc (Z), divided by the total number of drugs in the cluster.

All the structural calculations described above were performed for the extended simulated time, 2 μs - 4 μs.

Additionally, we calculated as a function of time (0 μs - 4 μs) the following:

- The **percentage probability of the peptides in β-sheet-like configurations** is the fraction of the number of peptides in β-sheet-like configurations within aggregated clusters, divided by the total number of peptides in the clusters. The results are presented as a function of time in a continuous moving average, calculated for time windows of 200 ns; e.g., the second point corresponds to the time average for time period 201-400 ns.

SM5 (D). Association free energy of drugs and peptides with the rest of the systems on the early-stage co-assembly and in ordered assemblies

The **association free energy** of a drug or a peptide with the rest of the system was calculated analogously to our previous paper[15], using Autodock4Zn[38]. These results were presented as a function of the **percentage ratio of solvent accessible surface area divided by the total surface area** per molecule (drug or peptide), which was calculated as follows. For each peptide and drug, we calculated the fraction of the solvent accessible surface area (SASA) of this as part of the system ($Å^2$) divided by the total surface area (TSA) ($Å^2$) of the same in the absence of the rest system. For all the solvent-accessible surface area calculations, we used Wordom [10,11,39] and the probe radius of IPA 2.5 Å[40,41].

These calculations were performed for representative early-stage co-assembled clusters per drug. We considered it important to define representative clusters as the ones that include peptides in β-sheet-like configurations, they are of high complexity (e.g., among the largest in size), they contain a sufficiently large number of drugs encapsulated, and they were not extracted from close-in-time simulation snapshots. Hence the selection was performed following the four criteria below, prioritized in a descending order: Clusters were selected (a) containing the highest number of peptides in β-sheet-like configurations per system, (b) having a size of no less than 20 molecules than the largest cluster per system, (c) containing at least 50% of the drugs encapsulated (i.e., 12 drug molecules or more), and (d) separated by time-window of at least 50 ns from each other.

Based on the above, the four most representative clusters selected per system were used for energy calculations ***(Figure 2A,B).*** Additionally, these calculations were performed for all molecules (drugs and peptides) within the ordered assemblies (as defined in SM4 (A)) within the final (100 ns) snapshot of simulations investigating FFWH ordered assemblies with different drugs, originating from the IPS scaffolds ***(Table S4, Sim. 4.113 – Sim. 4.120) (Figure 2 C,D).***

Table S4. Summary of the simulated systems mentioned in each supporting method section, and the aggregated simulation time

Methods Section	Description	N/A of simulation	Peptides/Scaffolds	Replicates	Time per Replicate (ns)	Aggregated Time (ns)
SM1	Screening-like simulations of minimalistic scaffolds	Sim. 1.1	NH_3^+-VFF-COO^-	x1	0-50	200
		Sim. 1.2	NH_3^+-LVFF-COO^-	x1	0-50	
		Sim. 1.3	NH_3^+-KLVFF-COO^-	x1	0-50	
		Sim. 1.4	NH_3^+-KLVFFA-COO^-	x1	0-50	
SM2	Simulations of modified LVFH scaffolds with Orange G	Sim. 2.1 - Sim. 2.3	NH_3^+-LVFF-COO^-	x3	0-100	900
		Sim. 2.4 - Sim. 2.6	Ac-LVFH-$CONH_2$, in the presence of ions	x3	0-100	
		Sim. 2.7 - Sim. 2.10	Ac-LVFH-$CONH_2$ in the absence of ions	x3	0-100	
SM3	Simulations of early-stage self-assembly of pristine peptides	Sim. 3.1	Ac-VWWH-$CONH_2$	x1	0-1000	3000
		Sim. 3.2	Ac-WFWH-$CONH_2$	x1	0-1000	
		Sim. 3.3	Ac-FFWH-$CONH_2$ [(*)]	x1	0-1000	
SM4	Simulations of ordered assemblies of selected consensus peptides	Sim. 4.1-Sim. 4.14	YWWH x 7{EPI, DOX, MTX, MIT, 5FU, CPT, CP} x 2 {IPS, SES}	x1	0-100	12,600
		Sim. 4.15-Sim. 4.28	FWWH x 7{EPI, DOX, MTX, MIT, 5FU, CPT, CP} x 2 {IPS, SES}	x1	0-100	
		Sim. 4.29-Sim. 4.42	HWWH x 7{EPI, DOX, MTX, MIT, 5FU, CPT, CP} x 2 {IPS, SES}	x1	0-100	
		Sim. 4.43-Sim. 4.56	WWWH x 7{EPI, DOX, MTX, MIT, 5FU, CPT, CP} x 2 {IPS, SES}	x1	0-100	
		Sim. 4.57-Sim. 4.70	YYWH x 7{EPI, DOX, MTX, MIT, 5FU, CPT, CP} x 2 {IPS, SES}	x1	0-100	
		Sim. 4.71-Sim. 4.84	LWWH x 7{EPI, DOX, MTX, MIT, 5FU, CPT, CP} x 2 {IPS, SES}	x1	0-100	
		Sim. 4.85-Sim. 4.98	IWWH x 7{EPI, DOX, MTX, MIT, 5FU, CPT, CP} x 2 {IPS, SES}	x1	0-100	
		Sim. 4.99-Sim. 4.112	QWWH x 7{EPI, DOX, MTX, MIT, 5FU, CPT, CP} x 2 {IPS, SES}	x1	0-100	
		Sim. 4.113-Sim. 4.126	FFWH x 7{EPI, DOX, MTX, MIT, 5FU, CPT, CP} x 2 {IPS, SES}	x1	0-100	
	Simulations of early-stage self-assembly of selected consensus peptides	Sim. 4.127	Ac-WWWH-$CONH_2$ [1(**)]	x1	0-1000	5,000
		Sim. 4.128	Ac-QWWH-$CONH_2$ [2(**)]	x1	0-1000	
		Sim. 4.129	Ac-FFWH-$CONH_2$ [(*),3(**)]	x1	0-1000	
		Sim. 4.130	Ac-HWWH-$CONH_2$	x1	0-1000	
		Sim. 4.131	Ac-YWWH-$CONH_2$ [4(**)]	x1	0-1000	
SM5	Simulations of early-stage co-assembly of top consensus peptides	Sim. 5.1-Sim. 5.7	WWWH x 7{EPI, DOX, MTX, MIT, 5FU, CPT, CP}	x1	0-2000	56,000
		Sim. 5.8-Sim. 5.14	QWWH x 7{EPI, DOX, MTX, MIT, 5FU, CPT, CP}	x1	0-2000	
		Sim. 5.15-Sim. 5.21	FFWH x 7{EPI, DOX, MTX, MIT, 5FU, CPT, CP} [5(**)]	x1	0-2000	
		Sim. 5.16-Sim. 5.28	YWWH x 7{EPI, DOX, MTX, MIT, 5FU, CPT, CP}	x1	0-2000	
	Simulations of early-stage self-assembly of top consensus peptides	Sim. 5.29	Ac-WWWH-$CONH_2$ [1(**)]	x1	1000-2000	16,000
		Sim. 5.30	Ac-QWWH-$CONH_2$ [2(**)]	x1	1000-2000	
		Sim. 5.31	Ac-FFWH-$CONH_2$ [3(**)]	x1	1000-2000	
		Sim. 5.32	Ac-YWWH-$CONH_2$ [4(**)]	x1	1000-2000	
		Sim. 5.33	Ac-WWWH-COO^-	x1	0-2000	
		Sim. 5.34	Ac-QWWH-COO^-	x1	0-2000	
		Sim. 5.35	Ac-FFWH-COO^-	x1	0-2000	
		Sim. 5.36	Ac-YWWH-COO^-	x1	0-2000	
	Simulations of early-stage co-assembly of top consensus peptide	Sim. 5.37-Sim. 5.45	FFWH x 7 drugs {EPI, DOX, MTX, MIT, 5FU, CPT, CP} [5(**)]	x1	2000-4000	14,000

[(*)] same simulated system

[(**)] extension of the same simulated system

Supporting Results

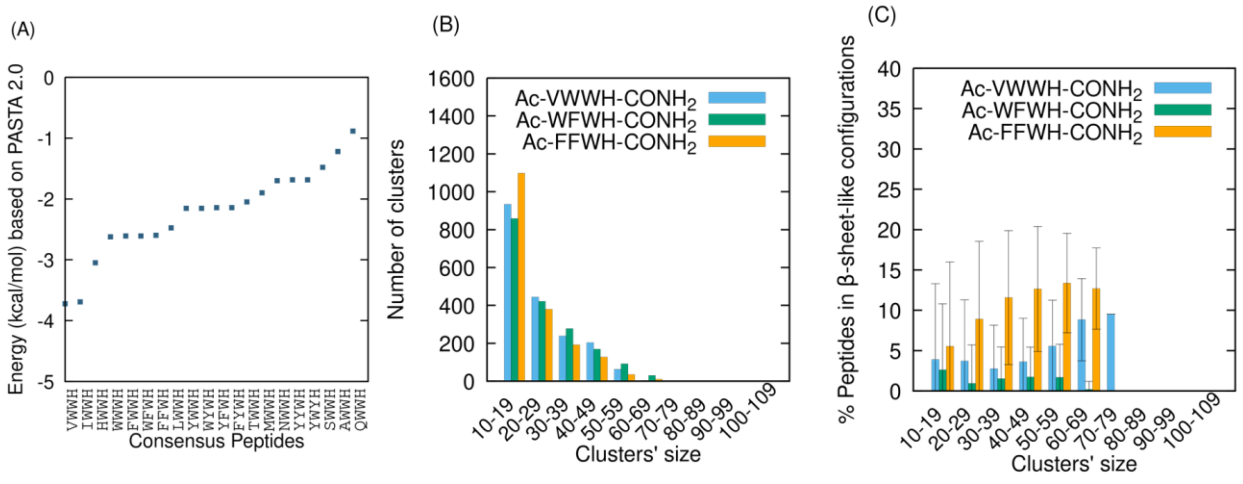

Figure S8. (A) Energy (kcal/mol) based on PASTA 2.0 for the top 20 consensus peptides based on the "consensus energetic penalty". (B) Number of clusters formed in the simulated systems consisting of Ac-VWWH-CONH$_2$ (light blue), Ac-WFWH-CONH$_2$ (green) and Ac-FFWH-CONH$_2$ (orange) as a function of clusters' size. (C) % Probability of Ac-VWWH-CONH$_2$ (light blue), Ac-WFWH-CONH$_2$ (green) and Ac-FFWH-CONH$_2$ (orange) to self-assemble in β-sheet-like configuration as a function of clusters' size. The large standard deviations can be attributed to the wide range of bin sizes and the absence of β-sheet formation in certain clusters.

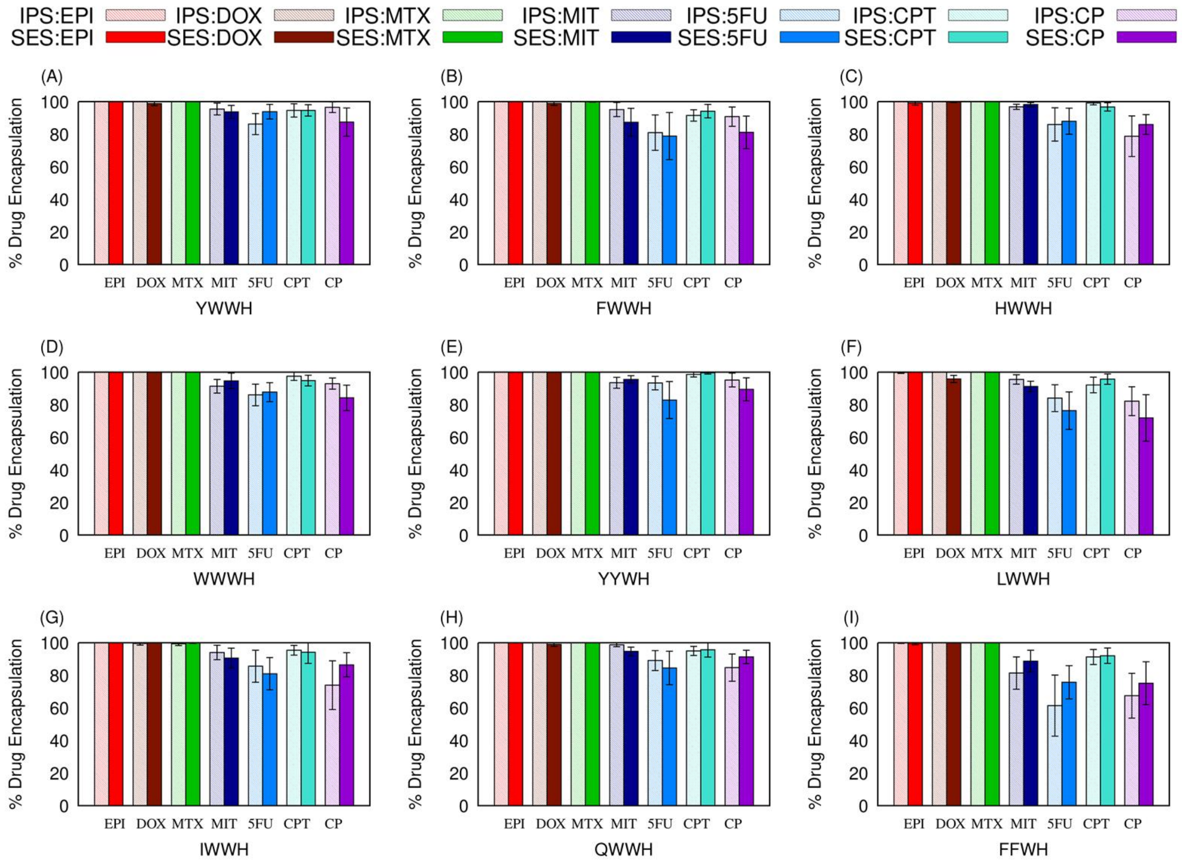

Figure S9. Average percentage of drug encapsulation in the pre-assembled scaffolds derived from both IPS scaffolds (solid bars) and SES scaffolds (dashed bars) for all the selected consensus peptides (A) YWWH, (B) FWWH, (C) HWWH, (D) WWWH, (E) YYWH, (F) LWWH, (G) IWWH, (H) QWWH and (I) FFWH in conjunction with the drugs under investigation (EPI-maroon, DOX-red, MTX-green, MIT-dark blue, 5FU-light-blue, CPT-cyan, CP-purple).

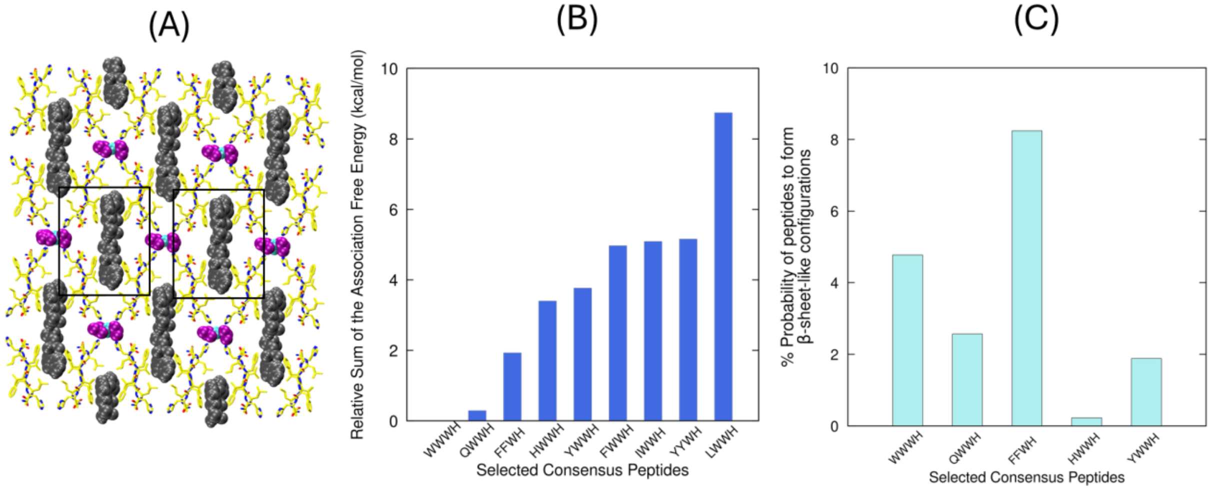

Figure S10. (A) Molecular graphics images[14] of the innermost pockets within ^{1}LVFH^{4}:Orange-G scaffold. The peptides are shown with yellow licorice representation, Orange-G with gray vdW, Zn^{2+} with cyan vdW and NO_3^- with purple vdW. (B) Relative Sum of the Association Free Energy (kcal/mol) for the selected consensus peptides (as defined in SM4(B)). (C) % Time average of peptides' probability to form β-sheet-like configurations.

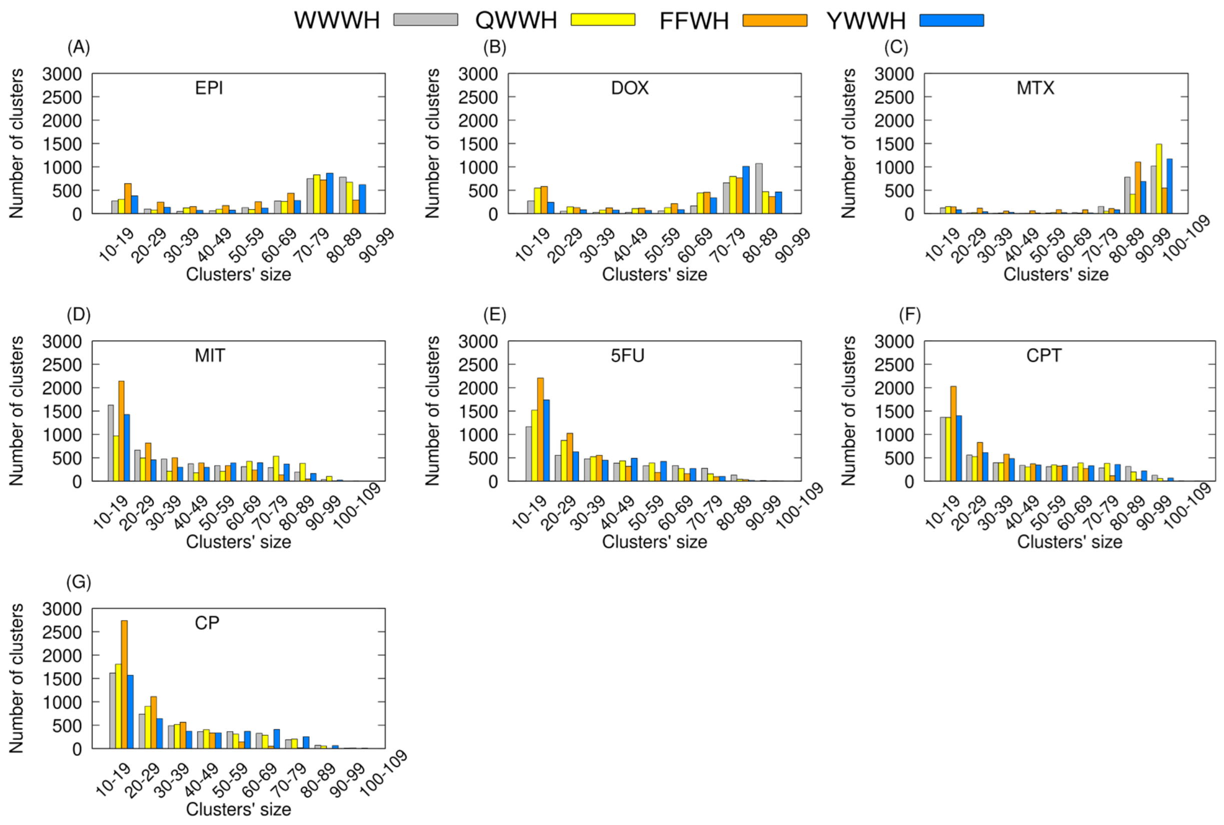

Figure S11. Number of formed clusters within the simulated systems composed by each one of the drugs under investigation (A) EPI, (B) DOX, (C) MTX, (D) MIT, (E) 5FU, (F) CPT, (G) CP along with each one of the peptides WWWH (gray), QWWH (yellow), FFWH (orange), and YWWH (blue) respectively.

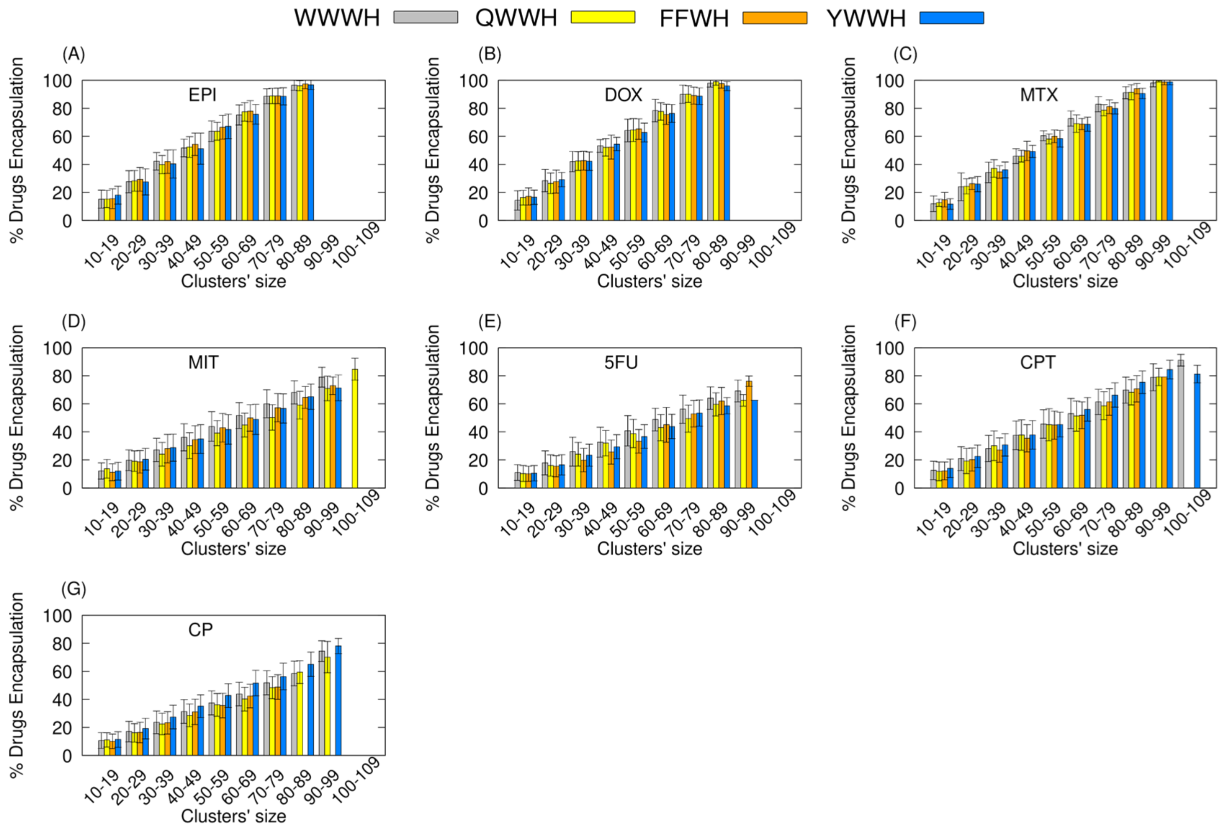

Figure S12. % Drug encapsulation by cluster size bin across clusters formed by (A) EPI, (B) DOX, (C) MTX, (D) MIT, (E) 5FU, (F) CPT, (G) CP, with each graph presenting data for the top peptides WWWH (gray), QWWH (yellow), FFWH (orange), and YWWH (blue) respectively.

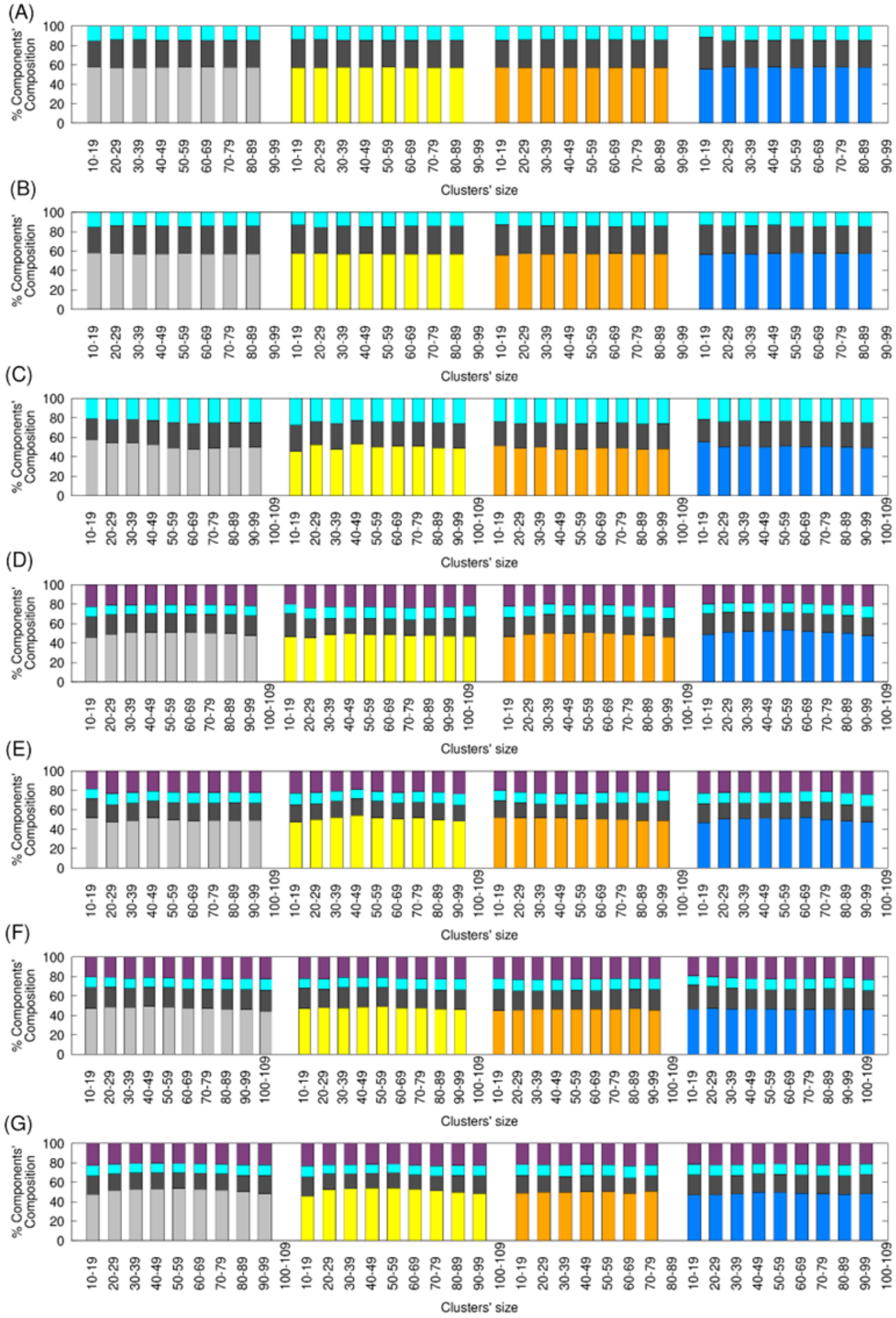

Figure S13. % Clusters' composition of all components: WWWH (gray), QWWH (yellow), FFWH (orange), YWWH (blue), drugs (gray), Zn^{2+} (cyan) and NO_3^- (violet) as a function of the clusters' size for clusters with: (A) EPI, (B) DOX, (C) MTX, (D) MIT, (E) 5FU, (F) CPT and (G) CP.

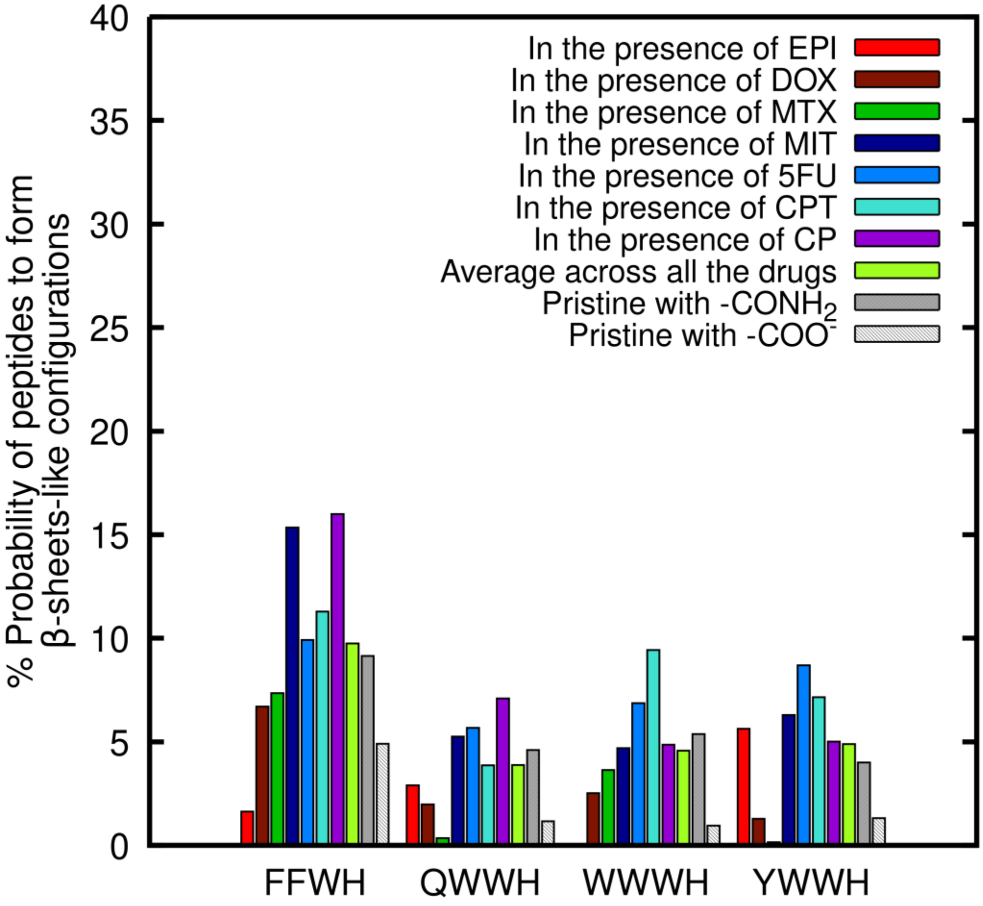

Figure S14. % Time average of peptides probability to form β-sheet-like configurations in the presence of EPI (red), DOX (maroon), MTX (green), MIT (dark blue), 5FU (light blue), CPT (turquoise), CP (light purple) and of the pristine peptides with -$CONH_2$ (gray) and -COO^- (dashed gray) terminals. The average of the time-weighted probability across the drugs is shown with light yellow.

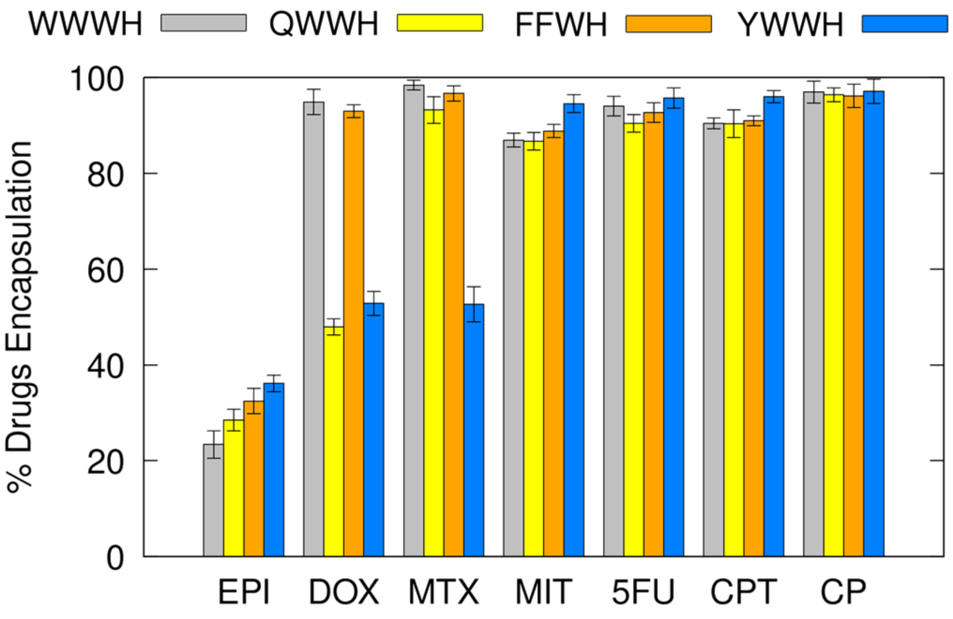

Figure S15. % Drug encapsulation experimentally assessed for the top consensus peptides YWWH (blue), FFWH (orange), WWWH (gray), and QWWH (yellow) across all the drugs under investigation: EPI, DOX, MTX, MIT, 5FU, CPT and CP, based on experiments.

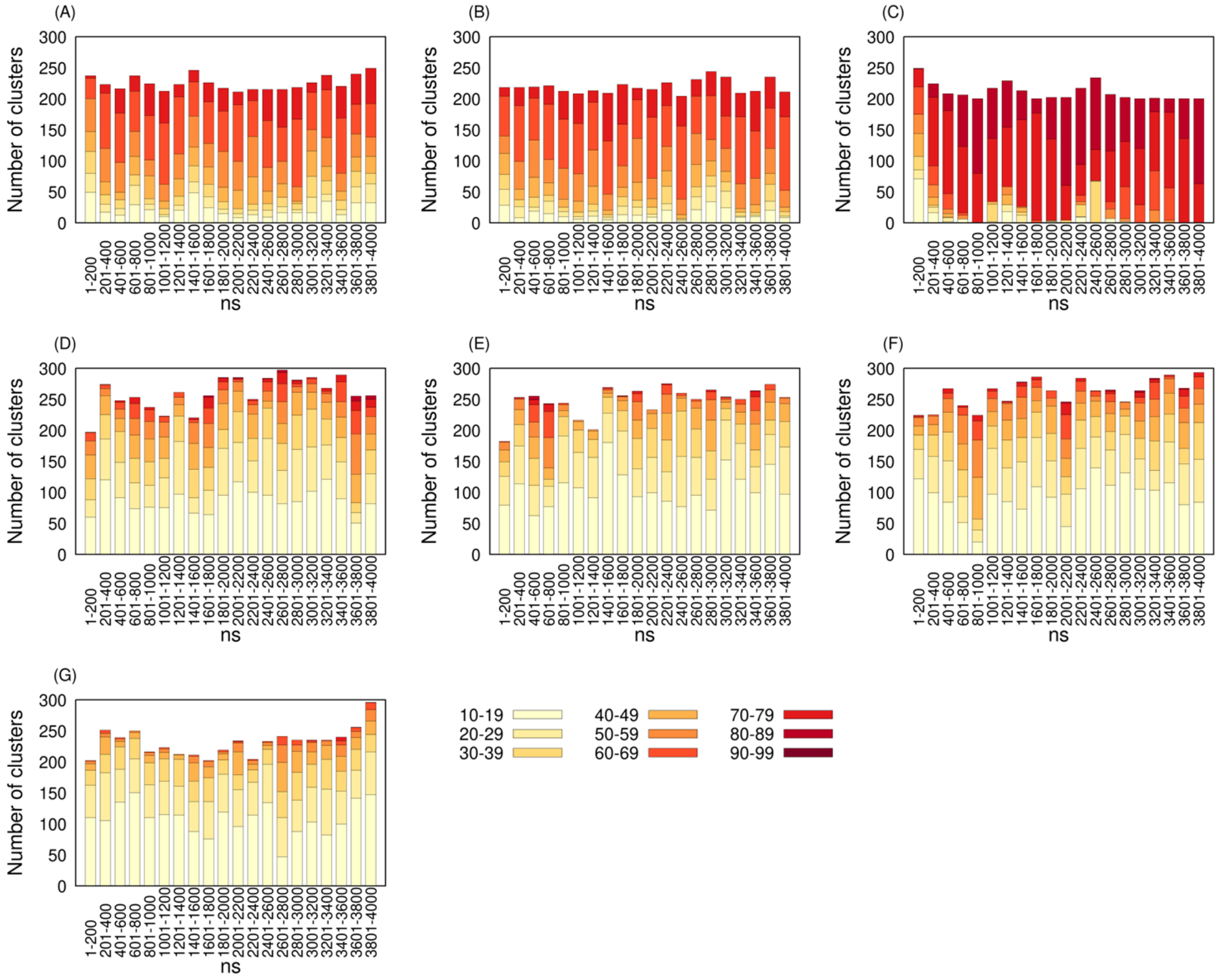

Figure S16. Number of clusters of different size classes as a function of consecutive 200 ns time windows in systems with (A) EPI, (B) DOX, (C) MTX, (D) MIT, (E) 5FU, (F) CPT, (G) CP.

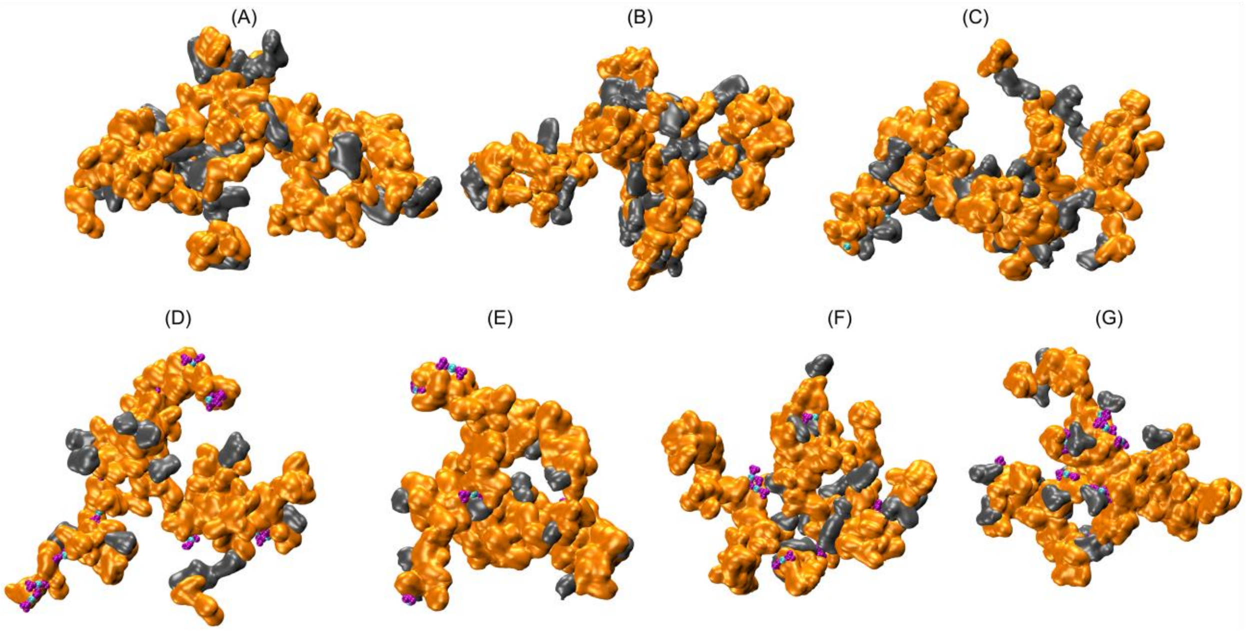

Figure S17. Molecular graphics images, using VMD[14], of the most representative clusters formed in MD simulations for each system including: (A) EPI, (B) DOX, (C) MTX, (D) MIT, (E) 5FU, (F) CPT and (G) CP. The FFWH peptides are shown in orange quick surface representation, the drugs in gray VDW representation, Zn^{2+} in cyan vdW representation, and NO_3^- in purple VDW representation.

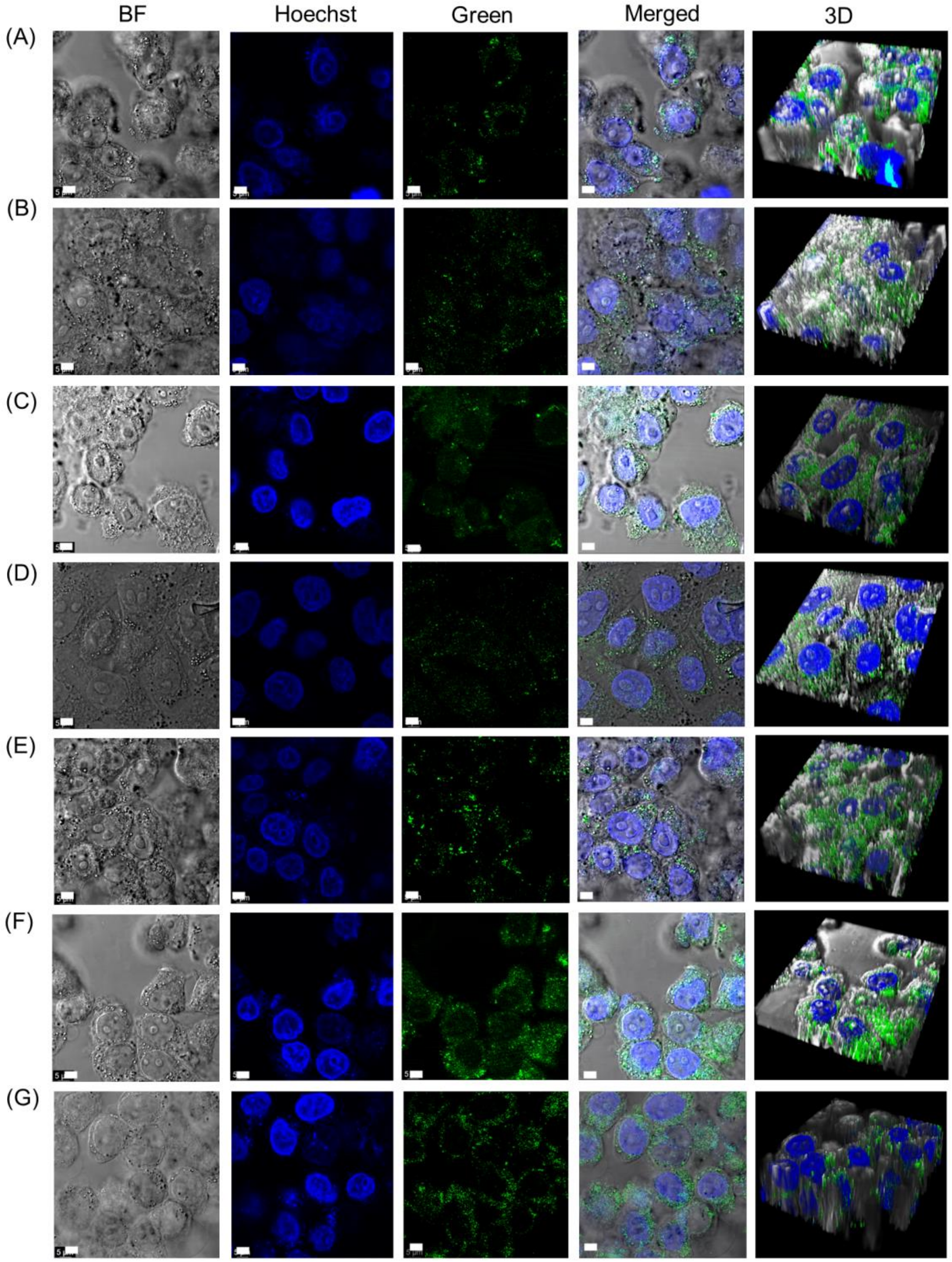

Figure S18. Live imaging of HeLa cells by confocal microscopy after a 24-hour incubation with (A) pristine EPI, (B) pristine DOX, (C) pristine MTX, (D) pristine MIT, (E) pristine 5FU, (F) pristine CPT, (G) pristine CP. The scale bar is 5 μm.

Supporting References

1 Landau M, Sawaya MR, Faull KF, Laganowsky A, Jiang L, Sievers SA, Liu J, Barrio JR, Eisenberg D. Towards a pharmacophore for amyloid. PLoS Biol. 2011;9(6):e1001080.

2 Pettersen EF, Goddard TD, Huang CC, Couch GS, Greenblatt DM, Meng EC, Ferrin TE. UCSF Chimera--a visualization system for exploratory research and analysis. J Comput Chem. 2004;25(13):1605-12.

3 Jo S, Kim T, Iyer VG, Im W. CHARMM-GUI: a web-based graphical user interface for CHARMM. J Comput Chem. 2008;29(11):1859-65.

4 Jo S, Cheng X, Islam SM, Huang L, Rui H, Zhu A, Lee HS, Qi Y, Han W, Vanommeslaeghe K, MacKerell AD Jr, Roux B, Im W. CHARMM-GUI PDB manipulator for advanced modeling and simulations of proteins containing nonstandard residues. Adv Protein Chem Struct Biol. 2014;96:235-65.

5 Park SJ, Kern N, Brown T, Lee J, Im W. CHARMM-GUI PDB Manipulator: Various PDB Structural Modifications for Biomolecular Modeling and Simulation. J Mol Biol. 2023;435(14):167995.

6 Kong L, Park SJ, Im W. CHARMM-GUI PDB Reader and Manipulator: Covalent Ligand Modeling and Simulation. J Mol Biol. 2024;436(17):168554.

7 Brooks BR, Brooks CL 3rd, Mackerell AD Jr, Nilsson L, Petrella RJ, Roux B, Won Y, Archontis G, Bartels C, Boresch S, Caflisch A, Caves L, Cui Q, Dinner AR, Feig M, Fischer S, Gao J, Hodoscek M, Im W, Kuczera K, Lazaridis T, Ma J, Ovchinnikov V, Paci E, Pastor RW, Post CB, Pu JZ, Schaefer M, Tidor B, Venable RM, Woodcock HL, Wu X, Yang W, York DM, Karplus M. CHARMM: the biomolecular simulation program. J Comput Chem. 2009;30(10):1545-614.

8 Lee J, Cheng X, Swails JM, Yeom MS, Eastman PK, Lemkul JA, Wei S, Buckner J, Jeong JC, Qi Y, Jo S, Pande VS, Case DA, Brooks CL 3rd, MacKerell AD Jr, Klauda JB, Im W. CHARMM-GUI Input Generator for NAMD, GROMACS, AMBER, OpenMM, and CHARMM/OpenMM Simulations Using the CHARMM36 Additive Force Field. J Chem Theory Comput. 2016;12(1):405-13.

9 Eastman P, Galvelis R, Peláez RP, Abreu CR, Farr SE, Gallicchio E, Gorenko A, Henry MM, Hu F, Huang J, Krämer A. OpenMM 8: molecular dynamics simulation with machine learning potentials. The Journal of Physical Chemistry B. 2023;128(1):109-16.

10 Seeber M, Cecchini M, Rao F, Settanni G, Caflisch A. Wordom: a program for efficient analysis of molecular dynamics simulations. Bioinformatics. 2007;23(19):2625-7.

11 Seeber M, Felline A, Raimondi F, Muff S, Friedman R, Rao F, Caflisch A, Fanelli F. Wordom: a user-friendly program for the analysis of molecular structures, trajectories, and free energy surfaces. Journal of computational chemistry. 2011;32(6):1183-94.

12 Tamamis P, Adler-Abramovich L, Reches M, Marshall K, Sikorski P, Serpell L, Gazit E, Archontis G. Self-assembly of phenylalanine oligopeptides: insights from experiments and simulations. Biophys J. 2009;96(12):5020-9.

13 Orr AA, Gonzalez-Rivera JC, Wilson M, Bhikha PR, Wang D, Contreras LM, Tamamis P. A high-throughput and rapid computational method for screening of RNA post-transcriptional modifications that can be recognized by target proteins. Methods. 2018;143:34-47.

14 Humphrey W, Dalke A, Schulten K. VMD: visual molecular dynamics. J Mol Graph. 1996;14(1):33-8, 27-8.

15 Vlachou A, Kumar VB, Tiwari OS, Rencus-Lazar S, Chen Y, Ozguney B, Gazit E, Tamamis P. Co-Assembly of Cancer Drugs with Cyclo-HH Peptides: Insights from Simulations and Experiments. ACS Appl Bio Mater. 2024;7(4):2309-2324.

16 Bhattacharyya R, Pal D, Chakrabarti P. Secondary structures at polypeptide-chain termini and their features. Acta Crystallogr D Biol Crystallogr. 2002;58(Pt 10 Pt 2):1793-802.

17 Krishna MM, Englander SW. The N-terminal to C-terminal motif in protein folding and function. Proc Natl Acad Sci U S A. 2005;102(4):1053-8.

18 Santiveri CM, Santoro J, Rico M, Jiménez MA. Factors involved in the stability of isolated beta-sheets: Turn sequence, beta-sheet twisting, and hydrophobic surface burial. Protein Sci. 2004;13(4):1134-47.

19 El-Agnaf OM, Bodles AM, Guthrie DJ, Harriott P, Irvine GB. The N-terminal region of non-A beta component of Alzheimer's disease amyloid is responsible for its tendency to assume beta-sheet and aggregate to form fibrils. Eur J Biochem. 1998;258(1):157-63.

20 Sciarretta KL, Boire A, Gordon DJ, Meredith SC. Spatial separation of beta-sheet domains of beta-amyloid: disruption of each beta-sheet by N-methyl amino acids. Biochemistry. 2006;45(31):9485-95.

21 Kern NR, Lee J, Choi YK, Im W. CHARMM-GUI Multicomponent Assembler for Modeling and Simulation of Complex Multicomponent Systems. bioRxiv [Preprint]. 2023:2023.08.30.555590. doi: 10.1101/2023.08.30.555590. Update in: Nat Commun. 2024;15(1):5459.

22 Putignano V, Rosato A, Banci L, Andreini C. MetalPDB in 2018: a database of metal sites in biological macromolecular structures. Nucleic Acids Res. 2018;46(D1):D459-D464. doi: 10.1093/nar/gkx989.

23 Andreini C, Cavallaro G, Lorenzini S, Rosato A. MetalPDB: a database of metal sites in biological macromolecular structures. Nucleic Acids Res. 2013;41(Database issue):D312-9.

24 Vainio MJ, Puranen JS, Johnson MS. ShaEP: molecular overlay based on shape and electrostatic potential. J Chem Inf Model. 2009;49(2):492-502.

25 Yoon K, Chen CC, Orr AA, Barreto PN, Tamamis P, Safe S. Activation of COUP-TFI by a Novel Diindolylmethane Derivative. Cells. 2019;8(3):220.

26 Jin UH, Park H, Li X, Davidson LA, Allred C, Patil B, Jayaprakasha G, Orr AA, Mao L, Chapkin RS, Jayaraman A, Tamamis P, Safe S. Structure-Dependent Modulation of Aryl Hydrocarbon Receptor-Mediated Activities by Flavonoids. Toxicol Sci. 2018;164(1):205-217.

27 Park H, Jin UH, Orr AA, Echegaray SP, Davidson LA, Allred CD, Chapkin RS, Jayaraman A, Lee K, Tamamis P, Safe S. Isoflavones as Ah Receptor Agonists in Colon-Derived Cell Lines: Structure-Activity Relationships. Chem Res Toxicol. 2019;32(11):2353-2364.

28 Cheng Y, Jin UH, Davidson LA, Chapkin RS, Jayaraman A, Tamamis P, Orr A, Allred C, Denison MS, Soshilov A, Weaver E, Safe S. Editor's Highlight: Microbial-Derived 1,4-Dihydroxy-2-naphthoic Acid and Related Compounds as Aryl Hydrocarbon Receptor Agonists/Antagonists: Structure-Activity Relationships and Receptor Modeling. Toxicol Sci. 2017;155(2):458-473.

29 Dayhoff MO, Schwartz RM, Orcutt BC. A model of evolutionary change in proteins. In: Dayhoff MO, editor. Atlas of protein sequence and structure. Silver Spring, MD: Nation Biomedical Research Foundation; 1978. pp. 345–52.

30 Jonnalagadda SVR, Gerace AJ, Thai K, Johnson J, Tsimenidis K, Jakubowski JM, Shen C, Henderson KJ, Tamamis P, Gkikas M. Amyloid Peptide Scaffolds Coordinate with Alzheimer's Disease Drugs. J Phys Chem B. 2020;124(3):487-503.

31 Jonnalagadda SVR, Kokotidou C, Orr AA, Fotopoulou E, Henderson KJ, Choi CH, Lim WT, Choi SJ, Jeong HK, Mitraki A, Tamamis P. Computational Design of Functional Amyloid Materials with Cesium Binding, Deposition, and Capture Properties. J Phys Chem B. 2018;122(30):7555-7568.

32 Kokotidou C, Jonnalagadda SVR, Orr AA, Vrentzos G, Kretsovali A, Tamamis P, Mitraki AA. Designer Amyloid Cell-Penetrating Peptides for Potential Use as Gene Transfer Vehicles. Biomolecules. 2019;10(1):7.

33 Quiroga R, Villarreal MA. Vinardo: A Scoring Function Based on Autodock Vina Improves Scoring, Docking, and Virtual Screening. PLoS One. 2016;11(5):e0155183.

34 O'Boyle NM, Banck M, James CA, Morley C, Vandermeersch T, Hutchison GR. Open Babel: An open chemical toolbox. J Cheminform. 2011;3:33.

35 Walsh I, Seno F, Tosatto SC, Trovato A. PASTA 2.0: an improved server for protein aggregation prediction. Nucleic Acids Res. 2014;42(Web Server issue):W301-7.

36 Lamiable A, Thévenet P, Rey J, Vavrusa M, Derreumaux P, Tufféry P. PEP-FOLD3: faster de novo structure prediction for linear peptides in solution and in complex. Nucleic Acids Res. 2016;44(W1):W449-54.

37 Frishman D, Argos P. Knowledge-based protein secondary structure assignment. Proteins. 1995;23(4):566-79.

38 Santos-Martins D, Forli S, Ramos MJ, Olson AJ. AutoDock4(Zn): an improved AutoDock force field for small-molecule docking to zinc metalloproteins. J Chem Inf Model. 2014;54(8):2371-9.

39 Buša J, Džurina J, Hayryan E, Hayryan S, Hu CK, Plavka J, Pokorný I, Skřivánek J, Wu MC. ARVO: A Fortran package for computing the solvent accessible surface area and the excluded volume of overlapping spheres via analytic equations. Computer Physics Communications. 2005;165(1):59–96.

40 Prakash P, Hancock JF, Gorfe AA. Binding hotspots on K-ras: consensus ligand binding sites and other reactive regions from probe-based molecular dynamics analysis. Proteins. 2015;83(5):898-909.

41 Bakan A, Nevins N, Lakdawala AS, Bahar I. Druggability Assessment of Allosteric Proteins by Dynamics Simulations in the Presence of Probe Molecules. J Chem Theory Comput. 2012;8(7):2435-2447.
